# Theory-guided design of S-doped Fe/Co dual-atom nanozymes for highly efficient oxidase mimics†

**DOI:** 10.1039/d4sc03101f

**Published:** 2024-08-16

**Authors:** Huan Cheng, Yanyue Chen, Mingjia Liu, Hongling Tao, Lu Chen, Fupeng Wang, Long Huang, Jian Tang, Tong Yang, Rong Hu

**Affiliations:** a College of Chemistry and Chemical Engineering, Yunnan Normal University Kunming Yunnan 650500 P. R. China hudierong_168@163.com yt09132149@163.com yangtong@ynnu.edu.cn; b Molecular Science and Biomedicine Laboratory, State Key Laboratory of Chemo/Biosensing and Chemometrics, College of Chemistry and Chemical Engineering, Collaborative Innovation Center for Molecular Engineering for Theronastics, Hunan University Changsha 410082 China; c National Engineering Research Center of Vacuum Metallurgy, Faculty of Metallurgy and Energy Engineering, Kunming University of Science and Technology Kunming Yunnan 650093 China tangjian9090@163.com

## Abstract

The advent of dual-atom nanozymes (DAzymes) featuring distinctive bimetallic active sites garnered significant attention, representing enhanced iterations of conventional single-atom nanozymes. The quest for an effective and universal strategy to modulate the catalytic activity of DAzymes posed a formidable challenge, yet few published reports addressed this. Herein, we designed and synthesized S-doped Fe/Co DAzymes (S-FeCo-NC) under theoretical guidance and revealed their excellent oxidase-like activity. Experimental and theoretical calculations indicated that the superior oxidase-like activity exhibited by S-FeCo-NC was attributed to the S-doping, which modulated the local electronic structure of the dual-atom active site. This modulation of the local electronic structure significantly optimizes oxygen adsorption energy, thereby accelerating the rate of enzyme-catalyzed reactions. As a proof-of-concept, this study integrated S-FeCo-NC with the cascade inhibition reaction of acetylcholinesterase (AChE) to devise a sensitive analytical platform for detecting organophosphorus pesticides. This study paved the way for elucidating the correlation between the local electronic structure of the active site and enzyme activity, offering novel methodologies and insights for the rational design of DAzymes.

## Abstract 2

In this study, we synthesized S-doped Fe/Co dual-atom nanozymes under theoretical guidance. S doping and the synergistic effect of Fe/Co dual-atom sites optimize the electronic structure of the active site, thus enhancing its oxidase-like activity.

## Graphical abstract

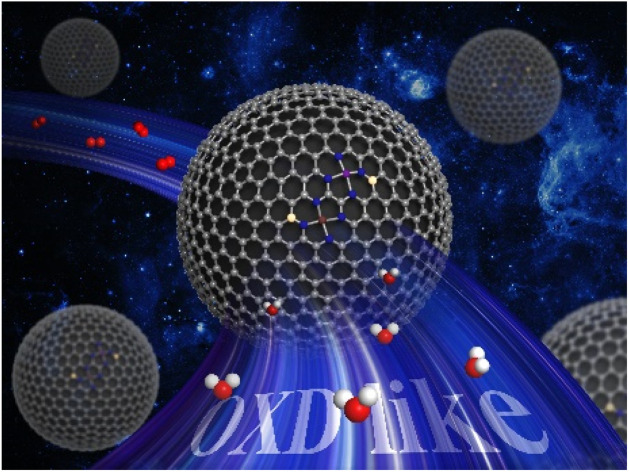

## Introduction

Single-atom nanozymes (SAzymes) garnered considerable attention and research interest as a novel class of nanozymes owing to their precisely defined atomic structure, optimal atom utilization efficiency, and ability to investigate catalytic mechanisms at the atomic scale.^1^ SAzymes with atomic dispersion could mimic a variety of natural enzymes with M–N_*X*_ as the catalytic center, such as oxidase (OXD),^2^ peroxidase (POD),^3^ catalase (CAT),^4^ superoxide dismutase (SOD),^5^*etc.* and are regarded as potential substitutes for natural enzymes. This well-defined and tunable active site center was an ideal model for understanding the structure–property relationship of enzymes and enzyme-like catalytic mechanisms, which contributed to the excellent application prospects of SAzymes in the fields of biosensing,^6^ biomedicine,^7^ and environmental protection.^8^ However, the symmetric electron distribution induced by the nonpolar M–N_4_ coordination structure of typical SAzymes resulted in undesirable adsorption strength of the catalytic reaction intermediates, which in turn affected the catalytic activity.^9^

Using dual-atom nanozymes (DAzymes) with isolated homonuclear/heteronuclear metal-atom pairs as active sites gained much attention from researchers as an effective improvement strategy.^10^ In contrast to single-atom nanozymes, there was a synergistic effect between the dual-atom sites in DAzymes, which provided additional adsorption sites and modulated the local electronic structure to obtain superior enzyme activity.^11^ Furthermore, the presence of neighboring pairs of metal atoms as active centers was expected to facilitate the emergence of novel catalytic mechanisms and enhance the adjustability of structure. Song *et al.*^12^ synthesized a dual-atom nanozyme Fe_1_Co_1_-NC in which the Co atomic site influenced the d-band center position of the Fe atomic site through synergistic effects and acted as a second reaction center, exhibiting excellent POD-like activity. Although similar reports made significant progress in the study of DAzymes, the catalytic activity of DAzymes still had a lot of room for improvement attributable to the unique homonuclear/heteronuclear dual-atom sites. Recent investigations unveiled that doping with low electronegativity non-metallic elements (such as B,^13^ S,^14^ P,^15^*etc.*) could effectively alter the coordination environment of central metal atoms, which represented a viable strategy for enhancing catalytic performance and stabilizing atomic structures. The presence of non-metallic heteroatoms can impact the local electronic structure surrounding the central metal atom, thereby altering its electron-withdrawing/donating properties and consequently modulating the kinetic activity of the M–N_4_ site.^16^ Li *et al.* presented a SAzyme with FeN_3_P as the active center (FeN_3_P-enzyme), wherein precise coordination of P and N modulated the electronic structure of the Fe active center, which demonstrated POD-like activity comparable to that of natural enzymes. Therefore, the heteroatom doping strategy was expected to modulate the local electronic structure of the dual-atom sites of DAzymes, aiming to optimize the adsorption strength of catalytic reaction intermediates and enhance enzymatic activity. Presently, research on DAzymes is in its nascent stages, with scarce reports of strategies involving the introduction of non-metallic heteroatoms around the dual-atom active site to enhance their catalytic activity, and the catalytic mechanism remained ambiguous. Moreover, while there were reports investigating the synergistic effects between dual-atom sites, few elucidated the mechanism underlying this synergy on catalytic activity at a deeper electronic level.

Herein, we report a theory-guided atomic site design strategy for the synthesis of DAzymes with S-doped Fe/Co dual-atom active sites on N-doped C substrates (S-FeCo-NC). Theoretical calculations were employed to screen two ideal dual-atom sites for OXD-like enzymes from the fourth-period transition metal elements (V, Cr, Mn, Fe, Co, Ni, and Cu). The doping of S atoms effectively modulated the coordination environment of the dual-atom site, leading to superior OXD-like activity surpassing that of most previously reported nanozymes. Experimental results and theoretical calculations demonstrated that this modulation of the local electronic structure induced an upward shift in the d-band center of the Fe site, enhancing the adsorption strength of oxygen intermediates and consequently improving the OXD-like activity of S-FeCo-NC. Furthermore, the interaction between the π orbitals of adsorbed O_2_ and the d orbitals of Fe resulted in the occupation of the π antibonding orbitals by electrons, thereby activating and elongating the O–O bond and accelerating the catalytic reaction process. The OXD-like activity of S-FeCo-NC surpassed that of Co-NC and Fe-NC by factors of 306 and 4.12, respectively. As a proof-of-concept, S-FeCo-NC was incorporated into a cascade inhibition reaction system involving AChE, enabling the sensitive detection of the chlorpyrifos pesticide with a detection sensitivity of 0.2 ng mL^−1^.

## Results and discussion

### Theory-guided design and synthesis of DAzymes

**Fig. 1:**
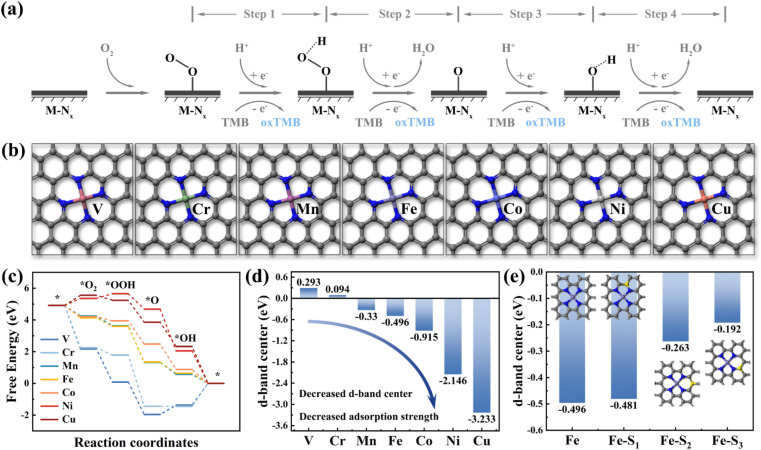
(a) Schematic diagram of the catalytic reaction mechanism of oxidase-like activity at the M–N_*X*_ site. (b) Modeling of M–N_4_ (M = V, Cr, Mn, Fe, Co, Ni, Cu) with different transition metals as central sites. (c) Step diagrams of adsorption energy changes and (d) d-band centers for seven M–N_4_ model oxidase-like catalytic reaction processes. (e) Histogram of d-band center changes for different S-doped sites.

Density Functional Theory (DFT) calculations are a method for exploring the electronic structures of multi-electron systems, frequently employed to investigate the physicochemical properties of molecules and atoms, and is one of the predominant research methodologies in physics, materials science, and computational chemistry.^17^ In this study, we utilized DFT calculations to screen two fourth-period transition metal elements as potential central atoms for dual-atom sites, aiming to advance the theory-guided synthesis of DAzymes. Previous studies have demonstrated that the OXD-like catalytic process follows a four-electron oxygen reduction reaction mechanism, emphasizing the metal site as the binding site for catalytic reactions.^18^ As illustrated in Fig. 1a, O_2_ was reduced to form two water molecules by acquiring four hydrogen protons and four electrons from the acidic buffer solution and TMB in the reaction while oxidizing TMB to blue oxTMB. The nanozymes catalyzing the complete reaction process were termed oxidative mimetic enzymes. Based on the active site characteristics of natural enzymes, we constructed seven structural models (M–N_4_) (M = V, Cr, Mn, Fe, Co, Ni, and Cu) with different metal atoms as the central sites (Fig. 1b). The adsorption strength of metal active sites for four oxygen-containing intermediates directly impacts the enzyme-catalyzed oxygen reduction reaction process.^19^ Excessive adsorption strength hampers intermediate desorption, thereby diminishing the reaction rate, whereas insufficient adsorption strength hampers intermediate adsorption at metal sites, hindering catalytic reactions.^20^ In the adsorption energy diagram depicted in Fig. 1c, manganese (Mn), iron (Fe), and cobalt (Co) demonstrated moderate adsorption strengths towards the four oxygenated intermediates, thereby exhibiting optimal theoretical OXD-like activities.

Moreover, the d-band center is highly correlated with the adsorption of small molecules on metal surfaces and has been effective as a descriptor for the catalytic activity of transition metal catalysts.^21^ As depicted in Fig. 1d, the d-band center of metal atoms decreased with increasing atomic number (decreased from 0.293 eV for Cr to −3.233 eV for Cu). This suggested a progressive decrease in the d-band center from left to right among transition metal elements within the same period and a gradual weakening of the adsorption strength for small molecules (Fig. S1†). The d-band centers of Mn, Fe, and Co were positioned centrally, consistent with the findings from the results of the adsorption energy step diagrams. These calculation results also agree with those reported earlier. Therefore, we selected Fe and Co, which exhibited similar atomic properties as the dual-atom sites for the nanozymes. Furthermore, the introduction of nonmetallic elements (such as B, S, P, N, O, *etc.*) to modulate the electronic structure of catalytically active sites has proven effective in enhancing the activity of nanozymes.^22–26^Fig. 1e illustrates that the d-band center of Fe atoms experienced varying degrees of elevation with the specific sites of sulfur doping. These calculations showed that doping of nonmetal atoms was expected to modulate the electronic structure of the catalytically active center to enhance the catalytic activity of the nanozymes.

**Fig. 2:**
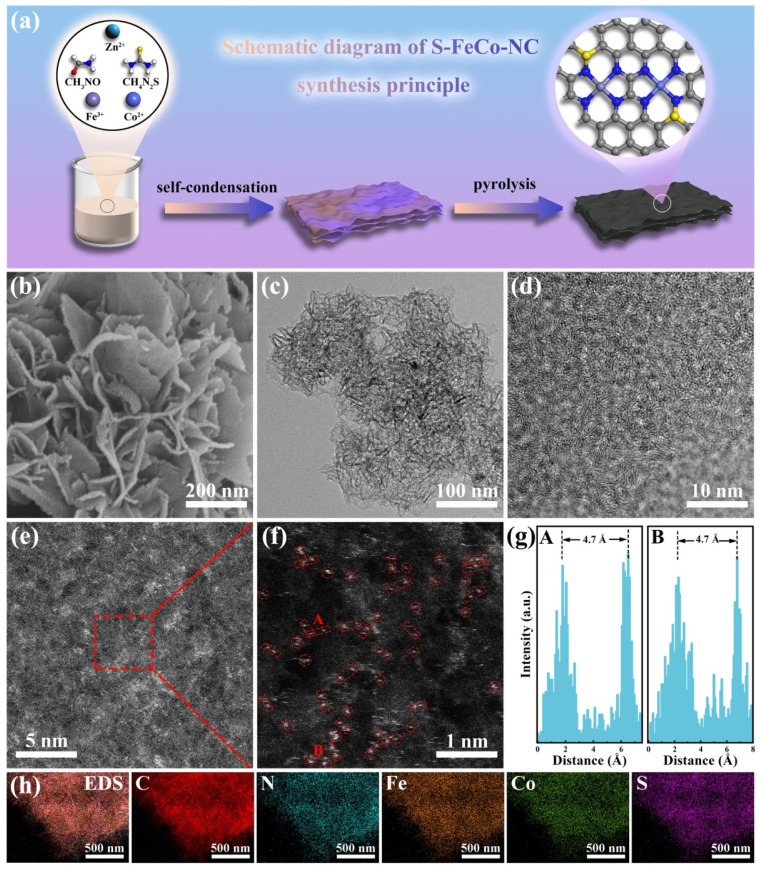
(a) Schematic diagram of the S-FeCo-NC synthesis process. (b) SEM, (c) TEM, and (d) HRTEM images. (e and f) AC-HAADF-STEM characterization images and Fe/Co dual-atom pairs are marked using red boxes. (g) Intensity distribution of dual-atom sites. (h) EDS mapping of S-FeCo-NC.

Building upon the above theoretical calculations, we successfully designed and prepared S-doped Fe/Co dual-atom nanozymes using a simple synthesis method. Fig. 2a illustrates the preparation process of S-FeCo-NC. Initially, transition metal ions and thiourea were introduced into a formamide (FA) solution to facilitate its self-condensation through a hydrothermal reaction, leading to the synthesis of the precursor. Formamide and thiourea molecules were used as C/N and S sources, respectively. Subsequently, the precursors underwent pyrolysis at 900 °C under argon to eliminate zinc atoms, which were used as a “grill” to prevent the accumulation of metal atoms. The high temperatures firmly anchored the metal atoms onto the sulfur-doped carbon nitride substrate, forming the final S-doped Fe/Co DAzyme. The Co SAzymes (Co-NC), Fe SAzymes (Fe-NC), and Fe/Co DAzyme (FeCo-NC) without S atoms were similarly prepared on N-doped C using an identical method.

### Characterization of materials

Scanning electron microscopy (SEM) (Fig. 2b) and transmission electron microscopy (TEM) (Fig. 2c) images elucidated the structural morphology of the S-FeCo-NC two-dimensional (2D) lamellar stack, revealing an absence of metal particles or clusters.^27^ The high-resolution transmission electron microscopy (HRTEM) image in Fig. 2d illustrated distorted, disordered, and discontinuous lattice streaks resulting from the migration of lattice atoms to interstitial positions induced by thermal fluctuations at elevated temperatures.^28^ Spherical aberration-corrected high-angle annular dark-field scanning transmission electron microscopy (AC-HAADF-STEM) was a pivotal characterization technique used to unveil the microstructure and atomic dispersion of materials. As shown in Fig. 2e, conspicuous large metal clusters were absent in the AC-HAADF-STEM image. The magnified image revealed a significant abundance of bright spots appearing in pairs, spaced at 4.7 ± 0.5 Å, tentatively affirming the existence of atomically dispersed Fe/Co dual-atom pairs on the carbon substrate (Fig. 2f and g).^29^ Inductively coupled plasma (ICP) was employed to characterize the actual content of the various elements in S-FeCo-NC. It revealed that 1.96 wt% Fe, 1.83 wt% Co, and 3.95 wt% S closely match the atomic proportions incorporated during synthesis (Fig. S2†). Furthermore, energy-dispersive X-ray spectroscopy (EDS) analysis of S-FeCo-NC confirmed the uniform distribution of Fe, Co, N, S, and C within the material (Fig. 2h).

**Fig. 3:**
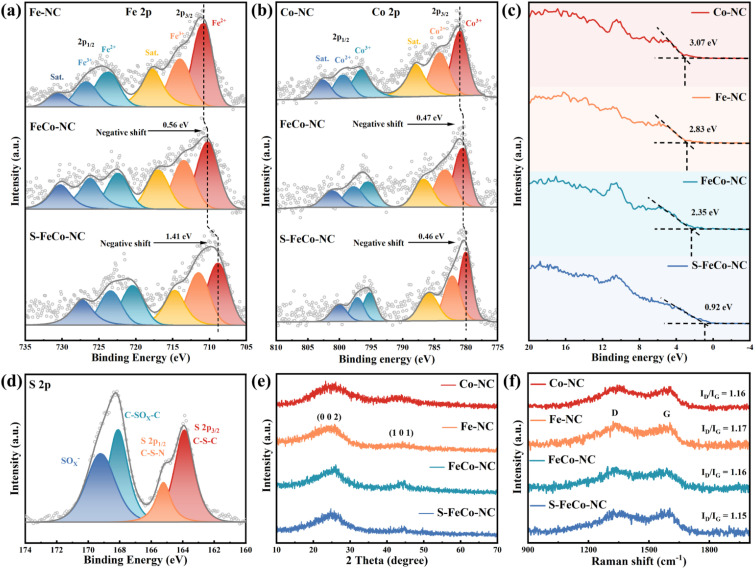
(a) Fe 2p spectra of Fe-NC, FeCo-NC and S-FeCo-NC. (b) Co 2p spectra of Co-NC, FeCo-NC and S-FeCo-NC. (c) Valence band spectra of all samples. (d) S 2p spectra of S-FeCo-NC. Superimposed (e) XRD and (f) Raman spectral profiles of four nanozymes.

X-ray photoelectron spectroscopy (XPS) further characterized the elemental composition and atomic valence of the material. Detailed integral fitting of the C 1s (Fig. S3†), N 1s (Fig. S4†), Fe 2p, Co 2p, and S 2p spectra was conducted using dedicated software.^30^ Additionally, thorough analysis and comparison of the fitted Fe 2p and Co 2p spectra were performed to elucidate the changes in the electronic structure of atoms. As depicted in Fig. 3a, the 2p_3/2_ binding energy peak position of elemental Fe in FeCo-NC experienced a negative shift from 710.81 eV in Fe-NC to 710.25 eV. Following S doping, the 2p_3/2_ binding energy peak position underwent an additional negative shift of 1.41 eV. It is noteworthy that a similar phenomenon of a gradual shift of the 2p_3/2_ peak to lower binding energies was observed when comparing Co 2p spectra (Fig. 3b). The UPS valence band spectrum not only elucidates the valence band structure of the material but also determines shifts in the d-band center.^31^ As shown in Fig. 3c, S-FeCo-NC exhibited the lowest valence band value of 0.92 eV, positioning it closer to the Fermi energy level than Co-NC, Fe-NC, and FeCo-NC. Given that the valence electrons near the Fermi energy level were mainly contributed by the d orbitals, this displacement of the valence band suggested a corresponding shift in the d-band center of the material. These findings suggested that S doping fully optimized the local electronic structure of the dual-atom sites, resulting in a negative shift in their binding energy, a reduction in the valence states of Fe and Co, and a shift of the d-band center. Moreover, these results validated the presence of synergistic effects between Fe and Co.^32–36^

The peak positions of the N elemental fine spectra were not significantly changed by S doping and the synergistic effects of dual-atom atoms.^37^ As depicted in Fig. S4,† N 1s fine spectra revealed a characteristic four-peak distribution corresponding to the M–N_4_ configuration, including pyridine-N (398.43 eV), pyrrole-N (399.76 eV), graphite-N (401.17 eV), and oxidized-N (404.38 eV). Pyridine-N was considered the primary anchoring localization site for metallic atoms. Distinctive peaks corresponding to C–S–C and C–S–N species were identified in the high-resolution S 2p spectra of S-FeCo-NC (Fig. 3d). The absence of elemental sulfur and metal–S bond signals suggested successful S-doping within the carbon substrate rather than being directly connected to the Fe/Co atoms.^38^ The X-ray diffraction (XRD) spectra of the four materials exhibited a similar peak structure (Fig. 3e), featuring characteristic peaks at 26.5° and 44.5° corresponding to the 002 and 101 crystal planes of graphitic carbon, respectively.^39^ No characteristic peaks of other metal oxides or metal particles were observed, which was consistent with the findings of morphological characterization. Raman spectroscopy of all samples exhibited D and G peaks originating from the carbon substrate at 1355 cm^−1^ and 1590 cm^−1^, with comparable *I*_D_/*I*_G_ values (Fig. 3f).^40^ This indicated that the four materials possessed a similar degree of graphitization, thereby excluding the effect of the carbon substrate on catalytic performance.

**Fig. 4:**
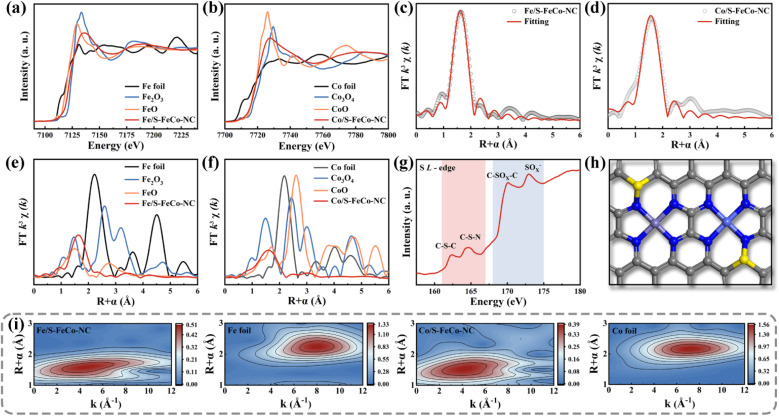
XANES spectrum of the (a) Fe and (b) Co K-edge. EXAFS of (c) Fe and (d) Co sites in S-FeCo-NC in *R*-space and the corresponding fitted curves. Comparative plots of (e) Fe and (f) Co sites with control samples in *R*-space. XANES spectrum of the (g) S L-edge. (h) The most probable atomic configuration of S-FeCo-NC. Color scheme: gray for C, dark blue for N, yellow for S, purple for Fe, and light blue for Co. (i) WT spectra of Fe, Co and control samples in S-FeCo-NC.

A synchrotron radiation X-ray source was utilized to characterize the X-ray absorption fine structure (XAFS) to investigate the electronic structure and atomic coordination environment of atoms in S-FeCo-NC, with a specific focus on the K-edge of Fe and Co and the L-edge of S.^41^ The absorption energies observed in the X-ray absorption near edge structure (XANES) spectra of elemental Fe (Fig. 4a) and Co (Fig. 4b) within S-FeCo-NC fell within the ranges of FeO to Fe_2_O_3_ and CoO to Co_3_O_4_, respectively. This suggested that the average oxidation states of Fe and Co ranged from +2 to +3 valence, consistent with findings from XPS analysis. The Fourier transform (FT) *k*^3^-weighted extended X-ray absorption fine structure (FT-EXAFS) spectra of Fe and Co exhibited two characteristic peaks at 1.6 Å and 1.58 Å belonging to the Fe–N and Co–N configurations, respectively (Fig. 4c and d). Compared with the control samples (Fig. 4e and f), no discernible characteristic peaks corresponded to Fe–Fe and Co–Co bonds beyond 2.5 Å, and no distinct Fe–Co peaks were identified (Fig. S5 and S6†). This suggested that Fe and Co predominantly existed in a single-atom state without direct interconnection, aligning with the characterization findings from AC-HAADF-STEM images. The EXAFS fitting data (Table S1 and S2†) indicated that each Fe or Co atom formed bonds with four N atoms, with an average bond length of 1.86 Å. This was similar to the structure of the M–N_*X*_ active site in the natural enzyme. Information on the three-dimensional arrangement of the metallic elements in *R* and *K* space was obtained through wavelet transform (WT) analysis. As illustrated in Fig. 4i, the contour plots exhibited maximum peaks for Fe and Co at approximately 4.1 Å^−1^ and 4.0 Å^−1^, attributed to the scattering of the Fe-N/Co-N species. The position of this intensity peak markedly differed from that of the peaks observed at 8 Å^−1^ for Fe and Co foil, as well as the double intensity peak observed for metal oxides (Fig. S7†). Moreover, the coordination environment of S in S-FeCo-NC merited attention (Fig. 4g), revealing distinct double peaks in the L-edge XANES spectra of S within the ranges of 161–167 eV and 168–175 eV. These peaks corresponded to the C–S–C and C–S–N coordinated species and the C–SO_*x*_–C and SO_*x*_^−^ coordinated species, consistent with the XPS characterization findings.^42–47^ Initial validation indicates direct bonding between S atoms and N/C.

**Fig. 5:**
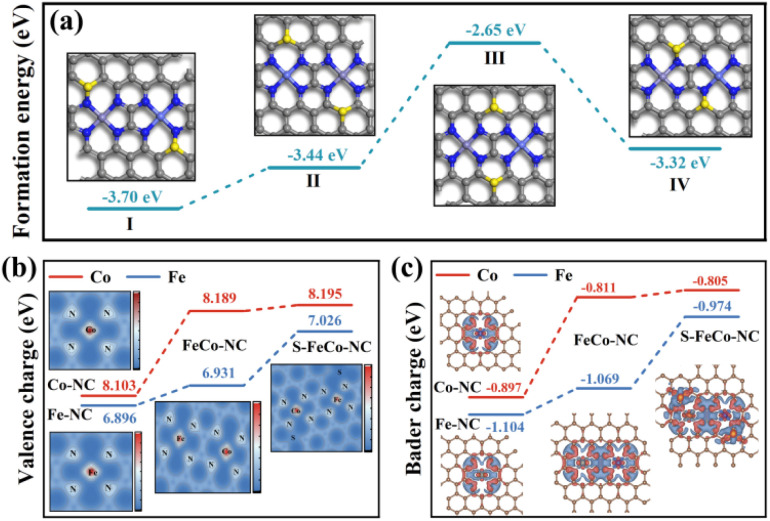
(a) DFT optimized models for different S atom doping positions and formation energies of the four conformations. (b) Valence charge densities (the inset shows a 2D charge density image) and (c) Bader charges of the four nanozymes (the inset shows a differential charge density plot and red indicates charge accumulation, while blue indicates charge depletion).

Drawing upon the characterization results above, this study employed DFT to calculate the bond length information for a series of dual-atom structure models to ascertain the actual configuration of the S-FeCo-NC active site (Fig. S8†). The calculations indicated that the metal–N bond lengths (1.86 Å) and dual-atom spacing (5.0 Å) depicted in the model presented in Fig. 4h aligned well with prior EXAFS fitting data and AC-HAADF-STEM characterization results, suggesting that this atomic structure was the most probable configuration for S-FeCo-NC. Moreover, four viable models were established to identify the precise doping sites of the S atoms (named I, II, III, and IV), and their respective formation energies were computed (Fig. 5a). I exhibited the lowest formation energy of −3.70 eV, suggesting that it was more thermodynamically favorable. The optimized atomic structure model reveals a direct bonding of S with N or C, which is consistent with XPS characterization and confirms precise S atom doping. The local electron distribution at the metal sites was investigated by computing charge density and Bader charge. As illustrated in Fig. 5b and c, the Fe and Co atoms within S-FeCo-NC exhibited the highest valence charge density (Fe 7.026 eV and Co 8.195 eV) and Bader charge (Fe −0.974 eV and Co −0.805 eV). Furthermore, the valence charge density (Fe 6.931 eV and Co 8.189 eV) and Bader charge (Fe −1.069 eV and Co −0.811 eV) of Fe and Co atoms in FeCo-NC were also markedly higher than those in Co-NC and Fe-NC. Increased valence electron densities and Bader charges suggested greater electronegativity at the metal sites, reduced likelihood of electron loss, and lower electronic valence states, consistent with the above XPS characterization results. This constituted compelling evidence that the doping of S and the synergistic effects between Fe and Co optimized the local electronic structure of the dual-atom sites.^48–52^

### Oxidase-like activity assays

**Fig. 6:**
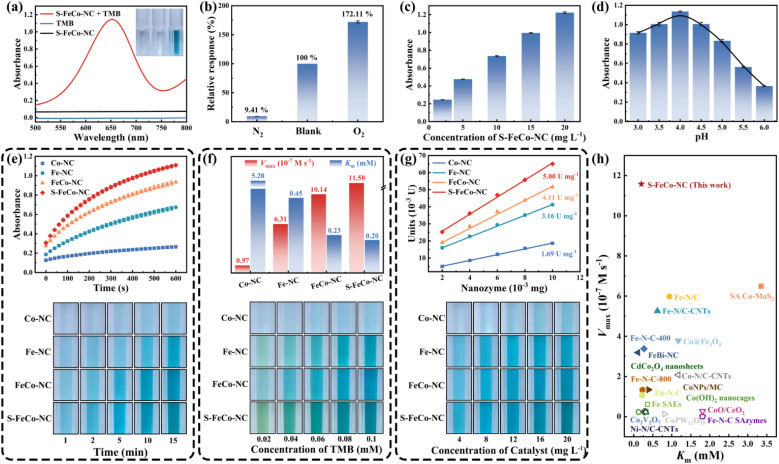
Evaluation of enzymatic activity of S-FeCo-NC. Enzymatic feasibility experiments were performed using (a) TMB as the chromogenic substrate. (b) The response of S-FeCo-NC in the N_2_ and O_2_ saturated states when TMB was the substrate. The absorbance of the reaction system at different (c) nanozyme concentrations and (d) pH values of buffer solution. (e) Enzyme kinetic activity profiles, (f) *K*_m_ and *V*_max_ histograms, (g) enzyme viability curves, and the corresponding pictures of solution color changes of Co-NC, Fe-NC, FeCo-NC, and S-FeCo-NC. (h) Evaluated in comparison to previously published standard kinetic activities of oxidase-like enzymes.

This study systematically compared and investigated the impact of different active sites on the OXD-like activity, utilizing 3,3′,5,5′-tetramethylbenzidine (TMB) as a substrate. As illustrated in Fig. 6a, the absorbance of the reaction system substantially increased in the presence of both TMB and S-FeCo-NC. In addition to TMB, comparable enhancements in absorbance were observed in reaction systems employing 2,2′-azinobis-(3-ethylbenzthiazoline-6-sulphonate) (ABTS) and *o*-phenylenediamine (OPD) as substrates (Fig. S9†). In the oxygen dependence experiment (Fig. 6b), the absorbance of the solution system increased to 172.11% of its initial value upon exposure to oxygen, contrasting sharply with the decrease to 9.41% of its initial value following the passage of N_2_ to eliminate dissolved O_2_ from the system. This evidence provided compelling support for the assertion that S-FeCo-NC exhibited OXD-like activity, which was significantly influenced by the presence of oxygen. Moreover, the absorbance of the reaction system exhibited an upward trend in correlation with the escalating concentration of S-FeCo-NC (Fig. 6c). As illustrated in Fig. 6d, S-FeCo-NC demonstrated optimal OXD-like activity under acidic conditions (pH = 4). This was attributed to the capacity of the acidic buffer solution to supply the requisite hydrogen protons for the spontaneous oxygen reduction reaction, amplifying the catalytic reaction rate. The kinetic profiles of enzyme-catalyzed reactions for the four nanozymes were compared over ten minutes, and absorbance images of the reaction systems were collected at various time points to evaluate their OXD-like activity (Fig. 6e). S-FeCo-NC demonstrated the highest OXD-like activity, followed by FeCo-NC, whereas Co-NC displayed the lowest enzyme activity. In standard steady-state kinetic assays employing TMB as a substrate (Fig. 6f and S10†), S-FeCo-NC displayed the lowest Michaelis constant (*K*_m_) and the highest enzymatic reaction rate (*V*_max_), measuring 0.20 mM and 11.58 × 10^−7^ M s^−1^, respectively.^53^ Its *K*_cat_/*K*_m_ values surpassed those of Co-NC by 310-fold, Fe-NC by 4.12-fold, and FeCo-NC by 1.31-fold. The results revealed that S-FeCo-NC exhibited a higher affinity and a greater catalytic rate towards TMB, surpassing the catalytic activity of most previously reported SAzymes (Fig. 6h and Table S3†). The specific activity is an important parameter for assessing the intrinsic catalytic activity of an enzyme. As depicted in Fig. 6g, S-FeCo-NC exhibited the highest specific activity at 5.0 U mg^−1^, followed by FeCo-NC at 4.11 U mg^−1^, surpassing Co-NC and Fe-NC at 1.69 and 3.16 U mg^−1^, consistent with prior kinetic experimental findings. Based on the experiments above, the ranking of OXD-like activities among the four nanozymes was as follows: S-FeCo-NC > FeCo-NC > Fe-NC > Co-NC. Furthermore, a series of tests were conducted on S-FeCoNC to assess its potential in practical applications. The findings demonstrated that all four prepared nanozymes exhibited excellent dispersibility in aqueous solutions, manifesting a distinct Tyndall effect under sunlight (Fig. S11a†). S-FeCo-NC retained its catalytic activity within the temperature range of 25–50 °C (Fig. S11b†), and the synthesized powders showed no significant decline in OXD-like activities over 30 days when stored at room temperature (Fig. S11c†).^54–57^ Furthermore, all five batches of S-FeCo-NC, synthesized at different times using an identical method, exhibited consistent powder properties, solubility, and oxidase-like activity. The XRD spectral peaks and specific activities of OXD-like enzymes also exhibited remarkable similarity (4.99 ± 0.0737 U mg^−1^) (Fig. S12 and S13†). These results indicate that S-FeCo-NC synthesized under theoretical guidance has excellent dispersion and stability, while the reproducibility of the experiments is outstanding and has good practical application.

### Theoretical evaluation of oxidase-like activity

DFT calculations were employed to further elucidate the modulation mechanism of the local electronic structure of the dual-atom sites by a precise S doping strategy. We conducted metal site closure experiments using KSCN as a blocking agent to confirm its role as an actual active center. As depicted in Fig. S14,† the OXD-like activity of S-FeCo-NC declined with increasing KSCN concentration, indicating that the dual-atom site served as the primary catalytic reaction site rather than the S-site or N-site.^58^

**Fig. 7:**
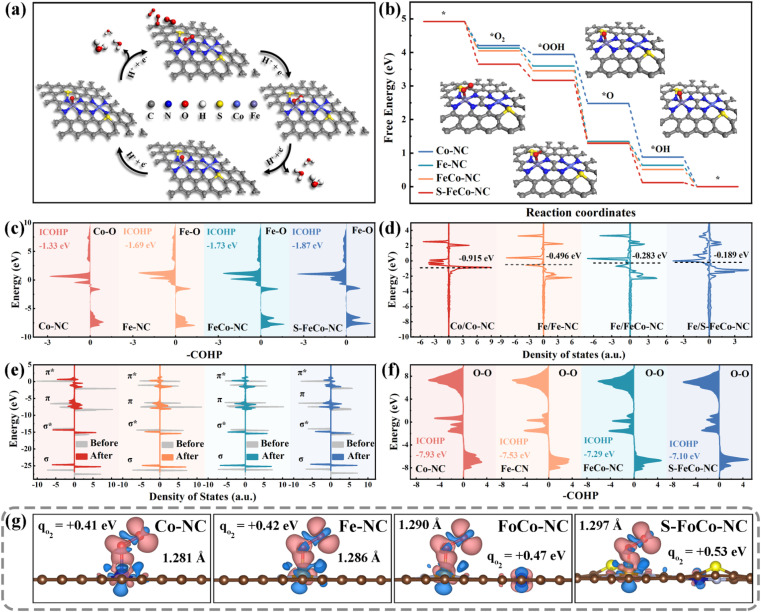
(a) Mechanisms of 4e^−^ pathway adsorption and desorption for the oxygen reduction reaction (ORR). (b) Step diagrams of adsorption energy changes of the four reaction intermediates on each of the four nanozymes. (c) COHP function between central atoms and adsorbed oxygen at different active sites. (d) Projected density of states of different active sites and d-band centers. (e) Comparative images of total density of states before and after adsorption of oxygen on different central atoms. (f) COHP function between oxygen atoms adsorbed at different active sites. (g) Three-dimensional charge density maps, charge transfer, and distance between O–O for Co-NC, Fe-NC, FeCo-NC, and S-FeCo-NC adsorbed oxygen intermediates (red indicates charge accumulation and blue indicates charge depletion).

The most stable adsorption configurations of the four oxygenated intermediates at the metal sites were calculated (Fig. S15†), and the specific pathway involving intermediate adsorption and desorption throughout the catalytic process was elucidated (Fig. 7a). The first step of O_2_ adsorption determined the following activity of electron transfer from active centers to adsorbed intermediates. Different catalytically active centers exhibited differing strengths of O_2_ adsorption, quantified by the calculated adsorption energy expressed as ΔG_*O_2__. As depicted in Fig. 7b, the alterations in the free energy of adsorption for the four reaction intermediates on various catalytically active sites were computed and presented using a step diagram. Evidently, S-FeCo-NC exhibited the highest ΔG_*O_2__ (1.27 eV), facilitating the catalytic reaction process due to this strong adsorption, resulting in superior OXD-like activity (Fig. S16†). Moreover, the adsorption energies for each of the four reactive intermediates at the Fe and Co sites were computed to identify the actual adsorption sites of the reactive intermediates. As illustrated in Fig. S17,† the ΔG_*O_2__ in S-FeCo-NC was greater at the Fe site than at the Co site. This indicated that the Fe site in S-FeCo-NC was more thermodynamically favorable for O_2_ adsorption, serving as the principal adsorption site for the catalytic reaction, whereas the Co site functioned as the secondary adsorption site (Fig. S18†). The bonding state between adsorbed O_2_ molecules and Fe atoms was assessed by calculating the crystal orbital Hamiltonian population (COHP) function. As depicted in Fig. 7c, peaks on the right correspond to bonding contributions, those on the left to antibonding contributions, and –COHP=0 signifies nonbonding contributions. The integral value ICOHP obtained by integrating the peaks below the Fermi energy level part could reflect the bond strength between two atoms to a certain extent. Clearly, S-FeCo-NC exhibited the lowest ICOHP value of −1.87 eV, suggesting the formation of more bonding orbitals between Fe atoms and adsorbed oxygen, thereby enhancing the Fe–O bond strength. This observation aligned with the earlier adsorption energy calculations. This improvement of the adsorption strength of the reaction intermediates stemmed from the upward shift of the d-band center caused by optimizing the local electronic structure of the catalytically active site. As illustrated in Fig. 7d, S-FeCo-NC, after local electronic structure optimization, possessed the highest d-band center at −0.189 eV, closer to the Fermi energy level than that of FeCo-NC (−0.283 eV), Fe-NC (−0.496 eV), and Co-NC (−0.915 eV). This finding aligned with the characterization data obtained from the UPS valence band spectrum.

Although the d-band center theory was widely acknowledged as a reliable gauge of the adsorption capacity of transition metal surfaces for small molecules, it simplified molecular energy levels and disregarded the occupied states of electrons within molecular orbitals. Hence, this study calculated the electron distribution within the molecular orbitals of adsorbed oxygen to further elucidate the effect of changes in the local electronic structure of the catalytic site on the catalytic activity. The comparison of total state densities of O_2_ molecules pre- and post-adsorption revealed that the density of states within the π orbitals exhibited marked splitting and dispersion compared to pre-adsorption (Fig. 7e). In contrast, the density of states within the σ orbitals experienced only minor shifts. The Bader charges and charge density difference were employed to quantify the electron gain and loss during oxygen adsorption (Fig. S19†). As illustrated in Fig. 7g, oxygen molecules adsorbed at different active sites received a certain number of electrons, and there was a concomitant increase in the O–O bond length. The electron-donating capacity of S-FeCo-NC experiences a substantial enhancement due to the modulation of its localized electronic structure. The oxygen molecule adsorbed on it achieved the highest electron transfer of 0.53 eV and formed the longest adsorbed O–O bond at 1.297 Å, considerably surpassing the free oxygen molecule (1.207 Å). These calculations proposed a robust interaction between the d orbitals of the central atom and the π orbitals of O_2_, resulting in the occupancy of the π antibonding orbitals of the adsorbed oxygen by electrons, thereby causing the O–O bond to become longer. Furthermore, the analysis of COHP was employed to extract bonding information regarding the adsorbed state O–O bonds. As depicted in Fig. 7f, S-FeCo-NC exhibited the highest ICOHP value of −7.10 eV, while FeCo-NC showed a slightly lower value of −7.29 eV, higher than the −7.93 eV of Co-NC and −7.53 eV of Fe-NC. This indicated that the bonding information of oxygen adsorbed on S-FeCo-NC exsited more antibonding orbitals contributions, resulting in a significant reduction in the O–O strength, which is in agreement with the previous calculations of the density of states. The activated O–O bond was more readily broken, thus enhancing both the reaction rate and the OXD-like activity. In summary, this paper provided a detailed elucidation of the regulatory mechanism for enhancing OXD-like activity due to the precise S doping and the synergistic effects of the dual-atom site, focusing on the local electronic structure, d-band centers, and molecular orbitals. The theoretical OXD-like activities, ranked from the highest to the lowest, were S-FeCo-NC > FeCo-NC > Fe-NC > Co-NC, consistent with experimental observations of enzyme catalytic performance.

### Detection of chlorpyrifos standards

**Fig. 8:**
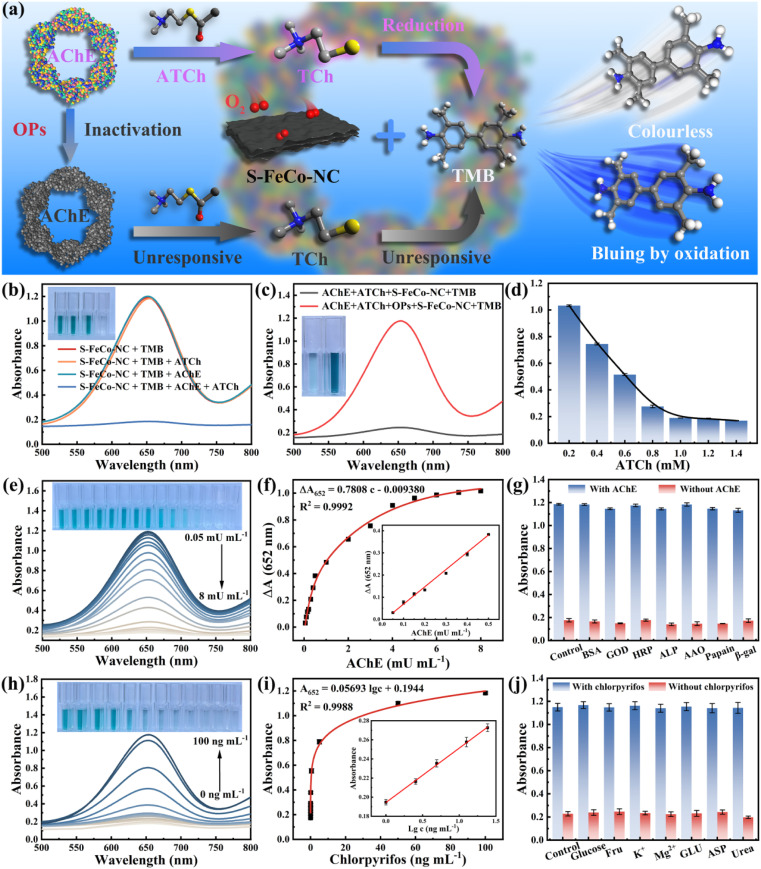
(a) Schematic diagram of the sensing platform of S-FeCo-NC/AChE/TMB for the detection of chlorpyrifos. Feasibility analysis of (b) enzyme inhibition reactions and (c) pesticide detection on the S-FeCo-NC/AChE/TMB platform. (d) Effect of different ATCh concentrations on the absorbance of the reaction system. (e) UV-vis absorption spectra and the corresponding color changes of the solution system in the presence of different concentrations of AChE. (f) The inset displays the linear correlation between the logarithm of AChE concentration and absorbance. (g) Colorimetric response of the solution system upon addition of interfering substances, with/without AChE. (h) UV-vis absorption spectra and the corresponding color changes of the sensor system in the presence of different concentrations of Chlorpyrifos. (i) The inset shows the linear relationship between the logarithm of Chlorpyrifos concentration and absorbance. (j) Colorimetric response of the solution system to interfering substances, with/without chlorpyrifos.

Organophosphorus pesticides (OPs), recognized as neurotoxins, kill harmful insects by irreversibly inhibiting acetylcholinesterase (AChE) activity within organisms. Nevertheless, unregulated and uncontrolled misuse of OPs could result in their accumulation throughout the food chain, posing significant risks to human life and health. The development of rapid and efficient methods for sensitively detecting OPs bore substantial implications for the global ecosystem and human health. As a proof-of-concept, this study integrated S-FeCo-NC with the cascade inhibition reaction of AChE to establish a sensitive sensing platform (S-FeCo-NC/AChE/TMB) for the sensitive detection of chlorpyrifos pesticides. Fig. 8a illustrates the schematic of the detection principle of the S-FeCo-NC/AChE/TMB sensing platform for chlorpyrifos detection. In the presence of AChE, acetylcholine underwent hydrolysis to form mercaptocholine, a potent reducing agent that hindered TMB oxidation, resulting in decreased absorbance of the solution system (Fig. 8b). Conversely, exposure to the chlorpyrifos pesticide led to AChE inactivation due to poisoning, thereby impeding mercaptocholine production and consequently increasing the absorbance of the solution system (Fig. 8c). The optimal reaction conditions for a reaction time of 15 minutes (Fig. S20†) and an ATCh concentration of 1 mM (Fig. 8d) were established to mitigate the impact of ATCh concentration and reaction time on the sensing detection platform.

As depicted in Fig. 8e, the absorbance of the solution system progressively declined with increasing AChE concentration, demonstrating excellent linearity within the range of 0.05 to 0.5 mU mL^−1^. The linear regression equation was Δ*A*_652_ = 0.7808*c* − 0.009380 (*R*^2^ = 0.9992, *n* = 7) (Fig. 8f), with a limit of detection (LOD) of 0.02 mU mL^−1^ (3*σ*/*S*), markedly lower by 1–2 orders of magnitude compared to that of the other assays outlined in Table S4.† Furthermore, this paper evaluated the influence of potential interferents on the detection platform, including papain, alkaline phosphatase, glucosidase, and so on. As illustrated in Fig. 8g, the S-FeCo-NC/AChE/TMB platform exhibited minimal susceptibility to these interferents, suggesting excellent selectivity and anti-interference capability. Due to the inhibitory effect of chlorpyrifos on AChE activity, the absorbance within the S-FeCo-NC/AChE/TMB system gradually increased with increasing chlorpyrifos concentration (Fig. 8h), displaying a favorable linear correlation within the range of 1 to 4 ng mL^−1^. The linear regression equation was *A*_652_ = 0.05693 lg *c* + 0.1944 (*R*^2^ = 0.9988, *n* = 7) (Fig. 8i), with a LOD of 0.2 ng mL^−1^ (3*σ*/*S*), surpassing the sensitivity of previously reported sensing systems (Table S5†). Similarly, this study investigated the anti-interference capability of the S-FeCo-NC/AChE/TMB detection platform by introducing seven substances as interferents, including glucose, metal ions, and urea. The findings revealed minimal color alteration in the S-FeCo-NC/AChE/TMB sensing platform under conditions of interference equivalent to 10 times the chlorpyrifos concentration, indicating its robust selectivity and anti-interference performance for chlorpyrifos detection (Fig. 8j). Furthermore, AChE and chlorpyrifos were each tested five times within five days using the developed sensing platform (Table S6 and S7†). The results demonstrate that the S-FeCo-NC/AChE/TMB sensing platform maintains high accuracy across varied time intervals, showing relative standard deviations of 2.10% and 4.74% over five assays and is aligned well with the linear equations (deviations of only 0.006 and 0.001). These results demonstrate that the sensing platform exhibits outstanding sensitivity and reproducible detection capabilities that are suitable for practical applications.^59–63^

### Detection of chlorpyrifos in an actual sample

To further evaluate the practical applicability and feasibility of the S-FeCo-NC/AChE/TMB sensing platform, chlorpyrifos residues in vegetables were detected utilizing the platform and retested using high-performance liquid chromatography (HPLC) to verify the accuracy of the results (Fig. S21†). Fig. S22† illustrates the growth of vegetables and the corresponding extract images. Due to the potential interference of the green color in the extract with the accuracy of colorimetric experiments, appropriate dilution measures were implemented to mitigate such interferences. Based on the derived linear regression equations, the results from three consecutive determinations after chlorpyrifos application yielded values of 195.5, 164.0, and 176.0 ng g^−1^, averaging 178.5 ng g^−1^, which was close to the value determined by high-performance liquid chromatography (182.5 ng g^−1^) (Table S8†). Furthermore, three distinct concentrations of chlorpyrifos solutions (1.5, 3, and 4 ng mL^−1^) were incorporated into the vegetable extracts for spiking recovery experiments (Table S9†). The outcomes revealed spiked recoveries of 102.00%, 99.67%, and 101.50%, with corresponding relative standard deviations of 2.67%, 3.02%, and 9.07%, respectively. These results collectively suggested that the S-FeCo-NC/AChE/TMB sensing platform exhibited exceptional sensitivity, selectivity, and anti-interference capabilities, and the detection accuracy rivaled that of commercial HPLC methods, and it showcased promising prospects for widespread practical applications.

## Conclusions

In summary, we reported a theory-guided approach for successfully screening, designing, and synthesizing S-doped Fe/Co dual-atom nanozymes, which exhibit superior oxidase activity compared to conventional SAzymes. Experimental and theoretical calculations revealed that S doping effectively modulated the local electronic structure of the dual-atom active site, leading to a substantial enhancement in the enzymatic activity of S-FeCo-NC. The modulation of the local electronic structure resulted in an elevation of the d-band center of the Fe active site and an augmentation in the adsorption strength of the adsorbed intermediate, thereby expediting the reaction kinetics. Moreover, the d orbital electrons at the active center occupied the π-antibonding orbitals of the adsorbed oxygen, resulting in the elongation of the O–O bond and consequently enhanced the catalytic reaction rate. The enzyme inhibition sensing platform constructed using S-FeCo-NC demonstrated remarkable sensitivity and selectivity in detecting the chlorpyrifos pesticide. This study offered a comprehensive and productive strategy for the systematic design and modulation of the catalytic activity of dual-atom nanozymes.

## Data availability

The relevant experimental and characterization data are available in the article and the ESI.†

## Author contributions

Huan Cheng: conceptualization, formal analysis, writing – review & editing. Yanyue Chen: data curation. Mingjia Liu: formal analysis. Hongling Tao: data curation, formal analysis. Lu Chen: investigation. Fupeng Wang: formal analysis. Long Huang: investigation. Jian Tang: methodology. Tong Yang; methodology. Rong Hu: conceptualization, writing – review & editing.

## Conflicts of interest

The authors declare no competing financial interest.

## Supplementary Material

SC-OLF-D4SC03101F-s001
