# Supplementary material for: Theory-guided design of S-doped Fe/Co dual-atom nanozymes for highly efficient oxidase mimics
Source: Chem Sci. 2024 Aug 16;15(36):14816–28. doi: 10.1039/d4sc03101f (PMC11342153; doi:10.1039/d4sc03101f)
Supplement: SC-015-D4SC03101F-s001 [file SC-015-D4SC03101F-s001.pdf]

Supplementary Information (SI) for Chemical Science.
This journal is © The Royal Society of Chemistry 2024

Electronic Supplementary Information

Theory-guided design of S-doped Fe/Co dual-atom nanozymes for highly efficient oxidase mimics

Huan Cheng, Yanyue Chen, Mingjia Liu, Hongling Tao, Lu Chen, Fupeng Wang, Long Huang, Jian Tang, Tong Yang*, Rong Hu**

College of Chemistry and Chemical Engineering, Yunnan Normal University, Kunming, Yunnan, 650500, P.R. China.

Rong. Hu

College of Chemistry and Chemical Engineering, Yunnan Normal University, Kunming, Yunnan, 650500, P.R. China.

Molecular Science and Biomedicine Laboratory, State Key Laboratory of Chemo/Biosensing and Chemometrics, College of Chemistry and Chemical Engineering, Collaborative Innovation Center for Molecular Engineering for Theronastics, Hunan University, Changsha, 410082, China.

Tong Yang

College of Chemistry and Chemical Engineering, Yunnan Normal University, Kunming, Yunnan, 650500, P.R. China.

Jian Tang

National Engineering Research Center of Vacuum Metallurgy, Faculty of Metallurgy and Energy Engineering, Kunming University of Science and Technology, Kunming, Yunnan, 650093, China.

* Corresponding author e-mail: hudierong_168@163.com, yt09132149@163.com, tangjian9090@163.com

1. Experimental

1.1. Materials

Formamide (FA ≥ 99%), zinc chloride ($ZnCl_2$ ≥ 98%), ferric chloride hexahydrate ($FeCl_3·6H2O$ ≥ 98%), cobalt chloride hexahydrate ($CoCl_2·6H_2O$ ≥ 98%), and thiourea (CH_4N_2S ≥ 98%) were purchased from Shanghai Aladdin Company. Sulfuric acid (H_2SO_4 ≥95%) was purchased from Shantian Pharmaceutical Co. All chemicals were standardized and not purified.

1.2. Preparation of S-FeCo-NC materials

In a typical synthesis method, the precursor of S-FeCo-NC was prepared by formamide self-condensation using a solvothermal method. 0.1 mol·L^{-1} of $ZnCl_2$, 0.005 mol·L^{-1} of $FeCl_3$ $6H_2O$, 0.005 mol·L^{-1} of $CoCl_2$ $_6H_2O$, and 0.005 mol·L^{-1} of CH_4N_2S were dissolved into 40 mL of formamide (FA), and ultrasonication for 30 min dissolved to obtain a homogeneous solution. The mixed solution was transferred to an autoclave and heated at 180 °C for 12 h. After cooling, the precursor was filtered and washed, purified three times using deionized water and ethanol, and dried under vacuum at 60 °C overnight. The dried precursor was then ground into a homogeneous powder and heated under the protection of argon gas flow at an elevated rate of 5 °C·min^{-1} to 900 °C and kept for 2 h. Finally, the powdered sample obtained by natural cooling was heat-treated in 0.5 M sulfuric acid at 80 °C for 8 h. The material was purified using water and ethanol until the pH value of the supernatant was 7 and vacuum-dried at 60 °C to obtain the final S-FeCo-NC.

1.3. Preparation of Co-NC, Fe-NC, and FeCo-NC materials

Similar to the preparation of S-FeCo-NC, Fe-NC was prepared without adding $CoCl_2·6H_2O$ and thiourea, and the addition of $FeCl_3·6H_2O$ was increased to 0.01 mol·L^{-1}. Co-NC was prepared without adding $FeCl_3·6H_2O$ and thiourea, and $CoCl_2·6H_2O$ was increased to 0.01 mol·L^{-1}. The preparation of FeCo-NC material was most similar to that of FeCo-NC but without adding thiourea.

2. Characterizations

Scanning electron microscope (SEM) images were recorded by a Gemini 300 thermal field emission

SEM. Transmission electron microscope (TEM) images were taken by FEI Tecnai G2 F30 manufactured by FEI, USA, with an accelerating voltage of 300 KV. High-angle annular dark-field scanning transmission electron microscope (HAADF-STEM) images and elemental mapping images were recorded by an FEI Tian G2 60-300 equipped with a spherical aberration corrector. Powder X-ray diffraction (XRD) patterns were recorded from 20° to 70° using a Japanese Rigaku Ultima IV at a scanning speed 2° min^{-1}. Raman spectra at 532 nm were recorded from 800-200 cm^{-1} using a LabRam HR Evolution Raman spectrometer. X-ray photoelectron spectra (XPS) were recorded using a Thermo ESCALAB 250 XI. Electron paramagnetic resonance (EPR) tests were performed on a Bruker EMX plus-6/1 spectrometer in Germany. The chemical composition content of the samples was analyzed using an inductively coupled plasma optical emission spectrometer (ICP-OES, Agilent 7700) and an organic elemental analyzer (Elemantar: Vario UNICUBE). The K-edge x-ray absorption spectra (XAS) of Fe, Co, and L-edge of S were collected at the Beijing Synchrotron Light Source for High Energy Physics (BSRT). The collected data were analyzed using Athena and Artemis software following standard procedures.

3. Evaluation of the oxidizing properties of catalysts

Oxidation activity was demonstrated by oxidizing 3,3′, 5,5′-tetramethylbenzidine (TMB) by turning blue. For this purpose, 4 mg of the prepared catalyst was dispersed in 10 mL of water and ultrasonically mixed thoroughly to obtain a catalyst suspension (400 $mg\cdot L^{-1}$). Then, 50 μL of catalyst suspension (400 $mg\cdot L^{-1}$) and 50 μL of TMB (2 mM) were added to 900 μL of HAc-NaAc buffer (0.1 M, pH 4). The absorbance at 652 nm (A652) was measured in kinetic mode with a Shimadzu UV-2700 spectrophotometer for 10 min. Relative activity was used as a criterion for evaluating the effect of various factors on the activity of the prepared catalyst-like oxidase.

4. Steady-state kinetic assays

The steady-state kinetic assays were determined as follows: 0.05 mL S-FeCo-NC (20 $mg\cdot L^{-1}$) and 0.05

mL TMB (final concentrations of 0.01 - 0.1 mM) were added to 0.9 mL HAc-NaAc buffer (0.1 M, pH 4). The above solutions were then incubated at 37 °C, and the A_{652} was measured at 15 min. The kinetic parameters were determined based on the equation

$$v = (V_{max} \times [S])/(K_m + [S])\#(1)$$

in which the v and V_{max} represent the initial and maximal reaction velocity, respectively, [S] is the substrate concentration, and K_m is the Michaelis constant.

The K_w value was calculated based on:

$$K_w = V_{max}/w\#(2)$$

where K_w stands for the TMB oxidation rate catalyzed by per unit mass concentration of nanozyme, and w is the mass concentration of catalyst.

5. Calculation of specific activity

The specific activity of the S-FeCo-NC was calculated using the method previously reported. Calculate the nanozyme activity (units) using the following equation (3) and calculate the specific activity (SA) of the nanozyme ($U \cdot mg^{-1}$) using the following equation (4):

$$b_{nanozymes} = V/(\varepsilon \times 1) \times (\Delta A/\Delta t)\#(3)$$

$$a_{nanozyme} = b_{nanozymes}/[m]\#(4)$$

where $b_{nanozymes}$ is the catalytic activity of nanozyme expressed in units. V is the total volume of reaction solution (μL). ε is the molar absorption coefficient of the colorimetric substrate, which is typically maximized at 39000 $M^{-1} \cdot cm^{-1}$ at 652 nm for TMB. 1 is the path length of light traveling in the cuvette (cm); A is the absorbance after subtraction of the blank value. ΔA/Δt is the initial rate of change in absorbance at 652 $nm \cdot min^{-1}$. $a_{nanozyme}$ is the SA expressed in units per milligram ($U \cdot mg^{-1}$) nanozymes, and [m] is the nanozyme weight (mg) of each assay.

6. Evaluation of acetylcholinesterase activity and its inhibitors

AChE activity was evaluated in the S-FeCo-NC/AChE/TMB system by adding 0.05 mL of 10 mM acetylthiocholine (ATCh) and 0.05 mL of AChE with different activities (0-80 mU·mL^{-1}) to 0.4 mL of Tris-HCl buffer (20 mM, pH 7.4). After incubation at 37 °C for 30 min, the reaction was stopped by adding 0.4 mL of HAc-NaAc buffer (0.1 M, pH 4). Subsequently, 0.05 mL of 0.4 g·L^{-1} of S-FeCo-NC and 0.05 mL of 2 mM TMB were added to the above mixture, and the characteristic absorbance was monitored at 652 nm after incubation at 37 °C for 15 min.

7. Interference resistance and selectivity

The selectivity and immunity to interference of the method for detecting AChE was assessed by adding a range of potential interfering substances, such as inorganic ions, small molecules, and enzymes, to the sensing system in the presence or absence of AChE.

8. Testing of chlorpyrifos standards

First, 0.05 mL of acetylcholinesterase (AChE) (70 mU·mL^{-1}) and 0.05 mL of different concentrations of chlorpyrifos standard solution was added to 0.35 mL of Tris-HCl buffer (20 mM, pH 7.4). After incubation at 37 °C for 30 min, 0.05 mL of 20 mM acetylthiocholine (ATCh) was added, and the reaction was incubated for an additional 30 min. 0.4 mL of HAc-NaAc buffer (0.1 M, pH 4) was added to stop the reaction. Subsequently, 0.05 mL of 0.4 g·L^{-1} of S-FeCo-NC and 0.05 mL of 2 mM TMB were added to the above mixture, and the characteristic absorbance was monitored at 652 nm after incubation at 37 °C for 15 min.

9. Meaning and calculation of error bars

The error bars displayed in all figures and the experimental data presented in this article, encompassing enzyme activity, feasibility, stability, pesticide detection, acetylcholinesterase detection, reproducibility, and Anti-interference test, were calculated from the standard deviations of three sets of parallel replicate experiments. The error bars depicted in the figures in this article were generated by plotting the standard deviation and mean as y-axis values. The formula is as follows:

$$S = \sqrt{\frac{\sum_{i=1}^{N}(X_i - \overline{X})^2}{n-1}}$$

$\overline{X}$ is the sample value, S is the standard deviation, and n is the number of samples.

10. Detection of chlorpyrifos in Brassica napus

The detection of chlorpyrifos in Chinese cabbage was carried out based on S-FeCo-NC/AChE/TMB system combined with an acetylcholinesterase cascade catalytic system, and the results were compared by high-performance liquid chromatography (HPLC). Firstly, the cabbages were divided into two groups. After the cabbages were well formed (about ten days), they were sprayed with 10 ml of chlorpyrifos solution (20 $mg \cdot L^{-1}$) every 2 days, three consecutively. The other group was not scattered with paraoxon and served as a control. Paraoxon residues in Chinese cabbage were extracted with CH_3OH by breaking 10 g of Chinese cabbage and then adding 10 mL of CH_3OH. In order to avoid the influence of cabbage color on the colorimetric test, the extract was centrifuged and filtered, and the supernatant was diluted 50-fold to obtain a colorless and transparent clarified solution for testing.

11. DFT calculations

The DFT calculation was carried out with Vienna Ab Initio Package. Using the generalized gradient approximation with Perdew–Burke–Ernzerhof formulation for the exchange-correlation potentials, the projected augmented wave potentials for the ionic cores, and a plane wave basis set with a kinetic energy cutoff of 500 eV for valence electrons. Partial occupancies of the Kohn–Sham orbitals were permitted using the Gaussian smearing approach and a width of 0.05 eV. The electronic energy could be considered self-consistent as the energy variance was below 10^{-5} eV. A geometry optimization was considered convergent as the force variance was below 0.02 $eV \cdot Å^{-1}$. Grimme's DFT-D3 method was utilized to explain the dispersion interactions. The $M\text{-}N_4$ (M = Fe, Co) centers were modeled on a (6*6) graphene monolayer supercell periodicity in the x and y directions, in which the S-FeCo-NC catalyst contained both $Fe\text{-}N_4$ and $Co\text{-}N_4$ sites.

A 15 Å vacuum layer along the vertical was set to avoid interactions between neighboring sheets. This graphene model contains 70 atoms. During structural optimiza-tions, the Γ point in the Brillouin zone was used for k-point sampling, and all atoms were allowed to relax fully. The free energy was calculated by the equation

$$G = E + ZPE - TS\#(5)$$

where E is the total energy, ZPE is the zero-point energy, T denotes absolute temperature and is 298.15 K, and S is the entropy. The adsorption energy Eads was defined as

$$E_{ads} = E_{total} - E_{substrate} - E_{molecule}\#(6)$$

where E_{total}, $E_{substrate}$, and $E_{molecule}$are the total energies of the substrate with adsorbate, clean substrate, and isolated adsorbate molecule, respectively.

The equations of O_2 reduction with four-electron pathway in the free energy profiles calculation are as follows:

(I) $*O_2 + H^+ + e^- = *OOH$

(II) $*OOH + H^+ + e^- = *O + H_2O$

(III) $*O + H^+ + e^- = *OH$

(IV) $*OH + H^+ + e^- = * + H_2O$

The asterisk (*) indicates the adsorption sites on a metal surface.

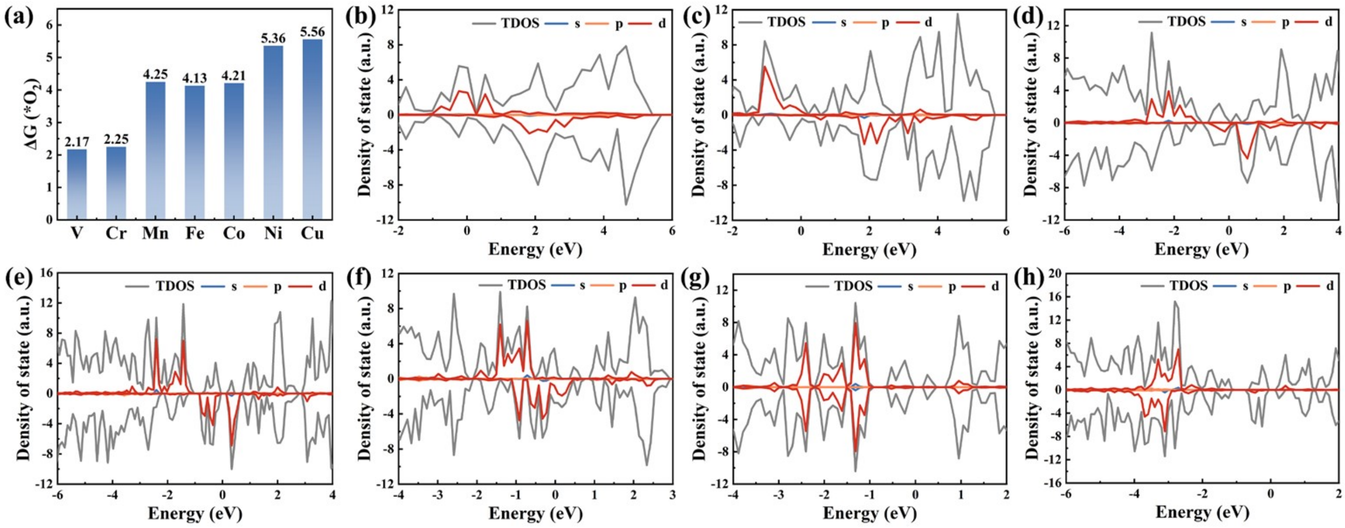

Figure S1. (a) A histogram illustrating the adsorption energies of oxygen molecules by oxidase-like models centered on diverse metal atoms. The density of states (DOS) of M-N_x materials centered on different metal elements. (b) V, (c) Cr, (d) Mn, (e) Fe, (f) Co, (g) Ni, and (h) Cu.

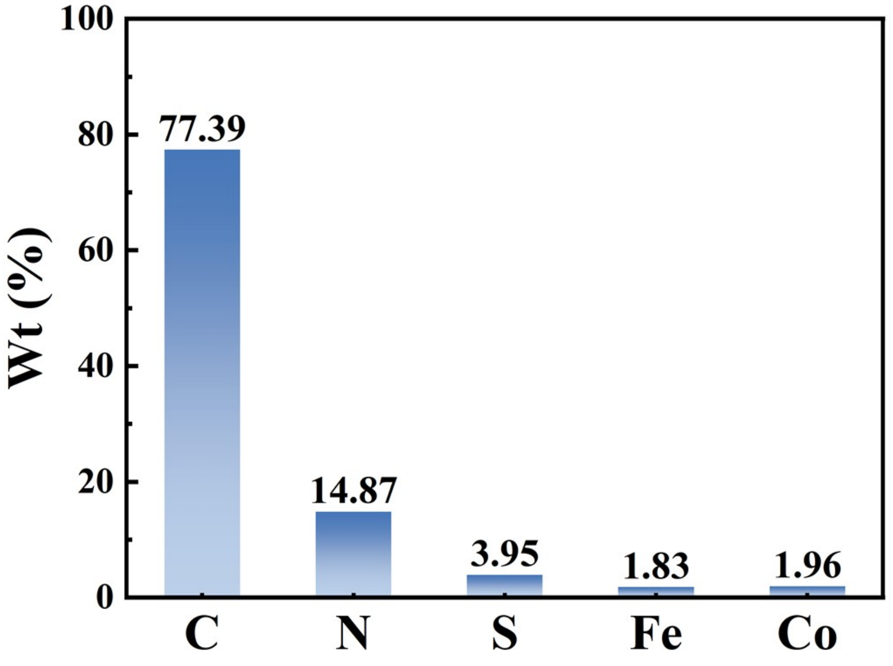

Figure S2. Histogram depicting the elemental content of S-FeCo-NC.

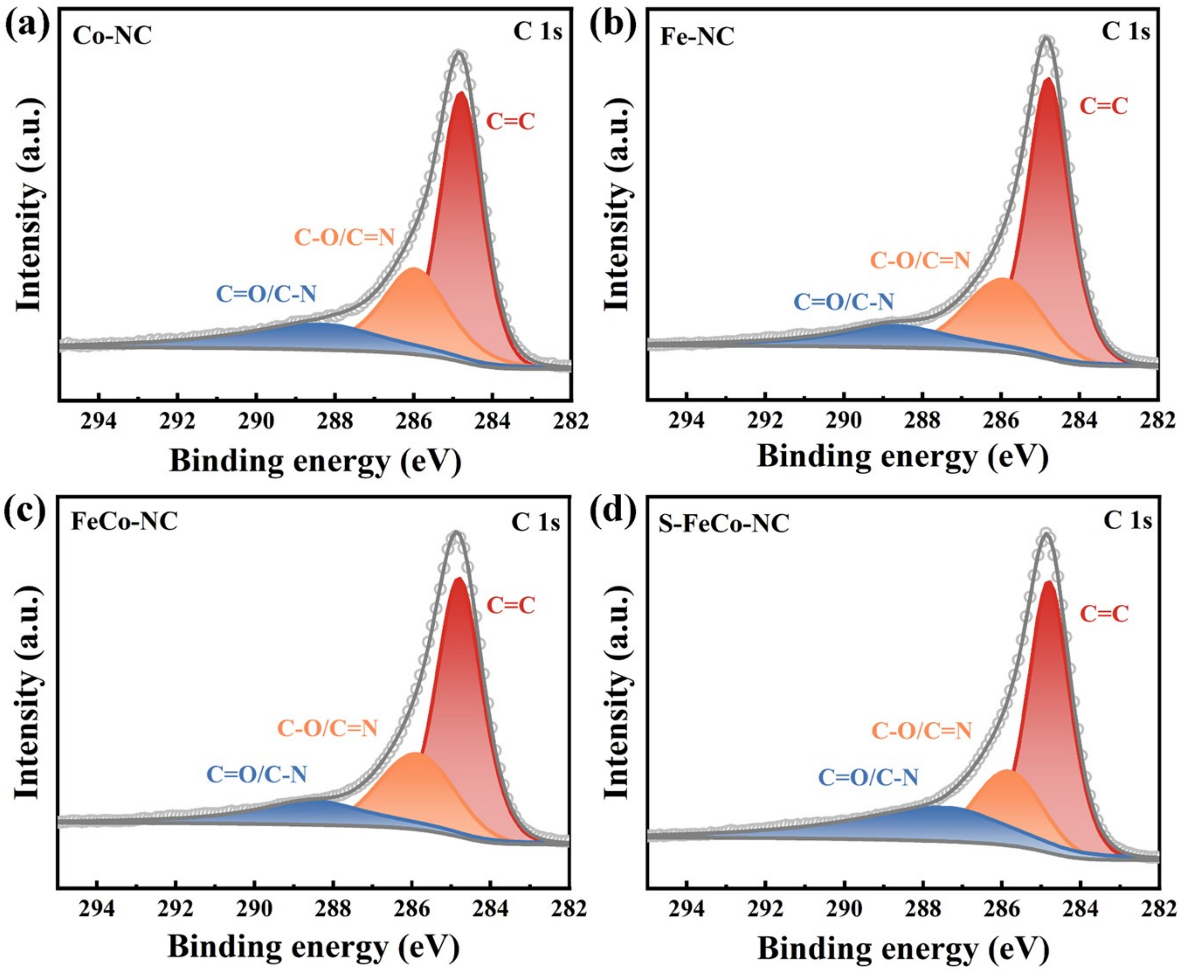

Figure S3. C 1s fitted spectral curves for (a) Co-NC, (b) Fe-CN, (c) FeCo-CN, and (d) S-FeCo-NC.

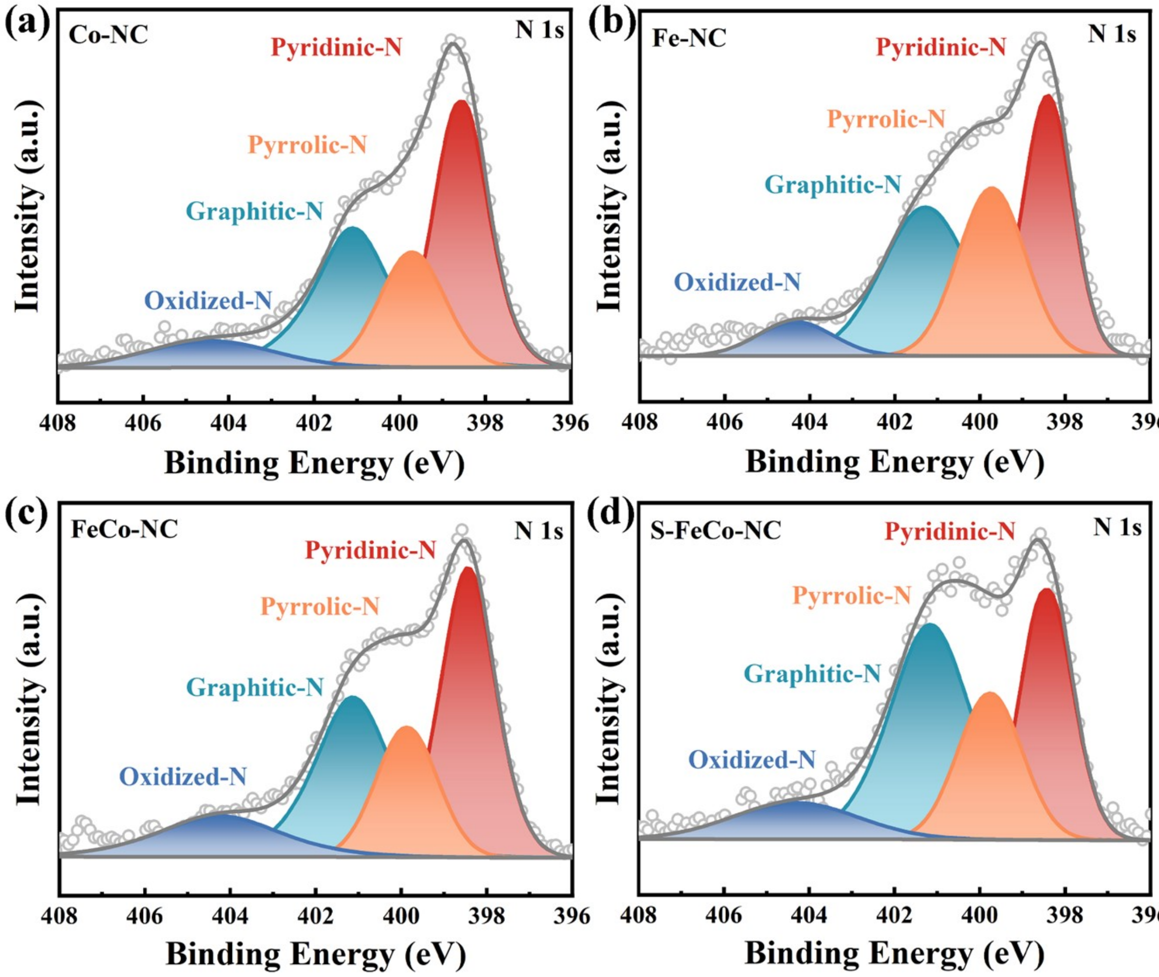

Figure S4. N 1s fitted spectral curves for (a) Co-NC, (b) Fe-CN, (c) FeCo-CN, and (d) S-FeCo-NC.

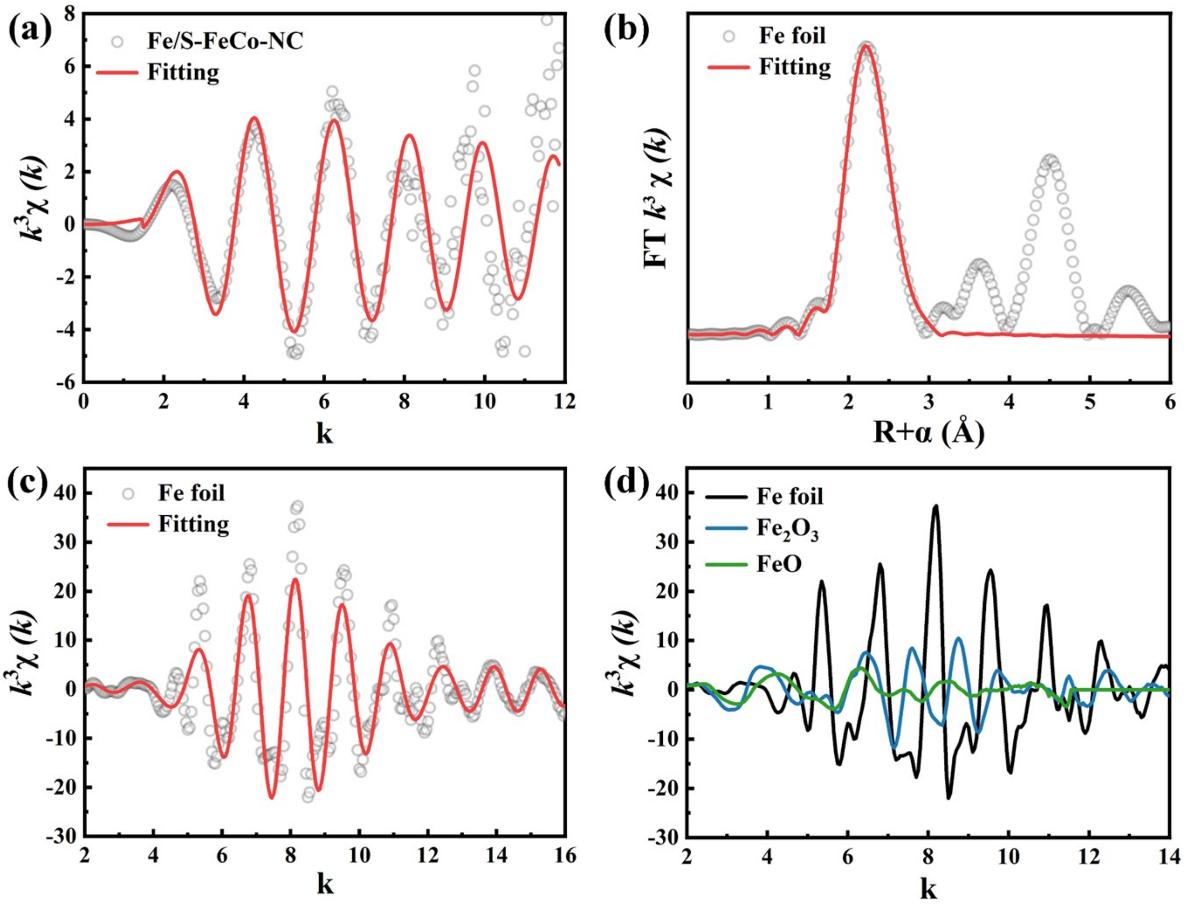

Figure S5. (a) Fourier-transformed (FT) -k^3-weighted χ(k)-function of the EXAFS spectra for Fe K-edge of S-FeCo-NC. (b) Fourier transform k^3-weighted EXAFS curves at Fe foil K-edge. (c) K-edge EXAFS fitting curves of Fe foil in k space. (d) K-edge EXAFS fitting curves of Fe foil, Fe_2O_3 and FeO in k space.

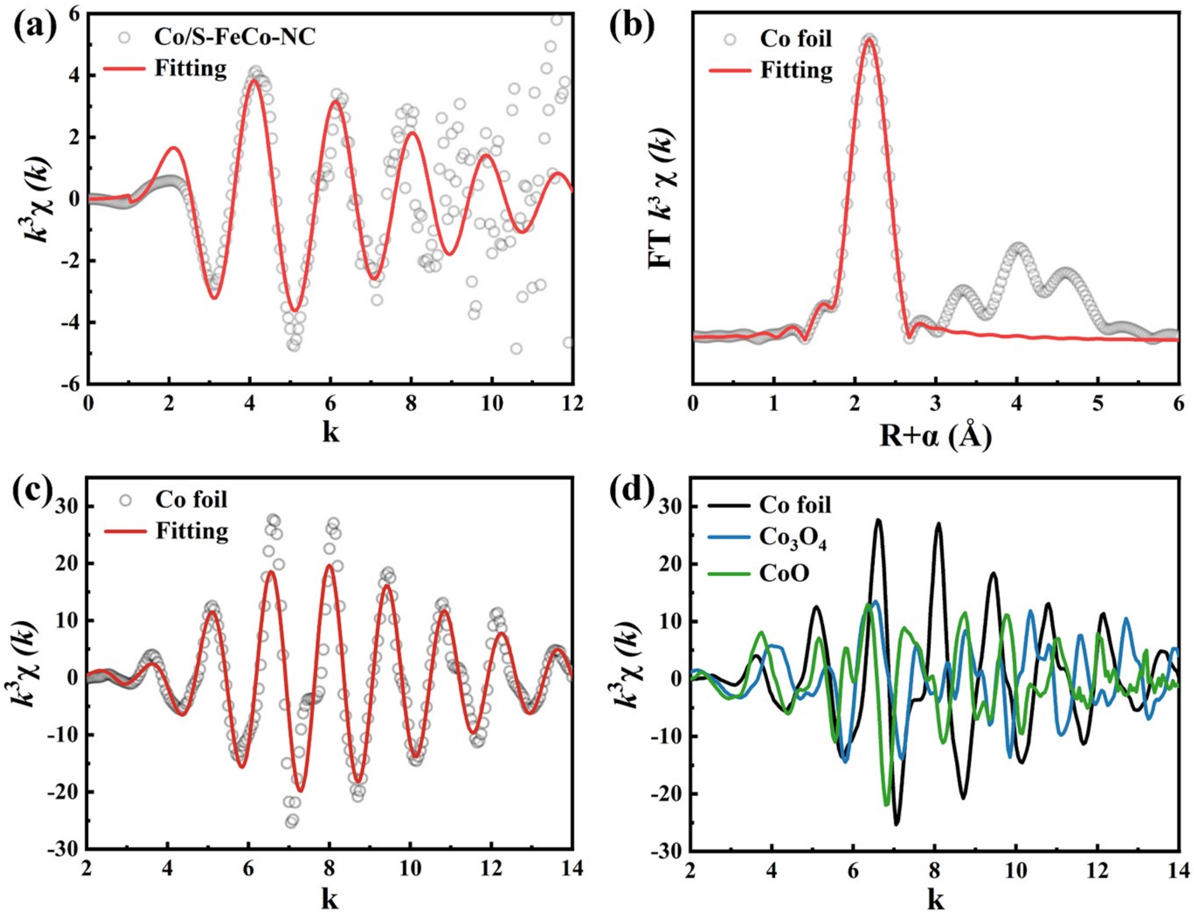

Figure S6. (a) Fourier-transformed (FT) -k^3-weighted χ(k)-function of the EXAFS spectra for Co K-edge of S-FeCo-NC. (b) Fourier transform k^3-weighted EXAFS curves at Co foil K-edge. (c) K-edge EXAFS fitting curves of Co foil in K-space. (d) K-edge EXAFS fitting curves of Co foil, Co_3O_4 and CoO in k space.

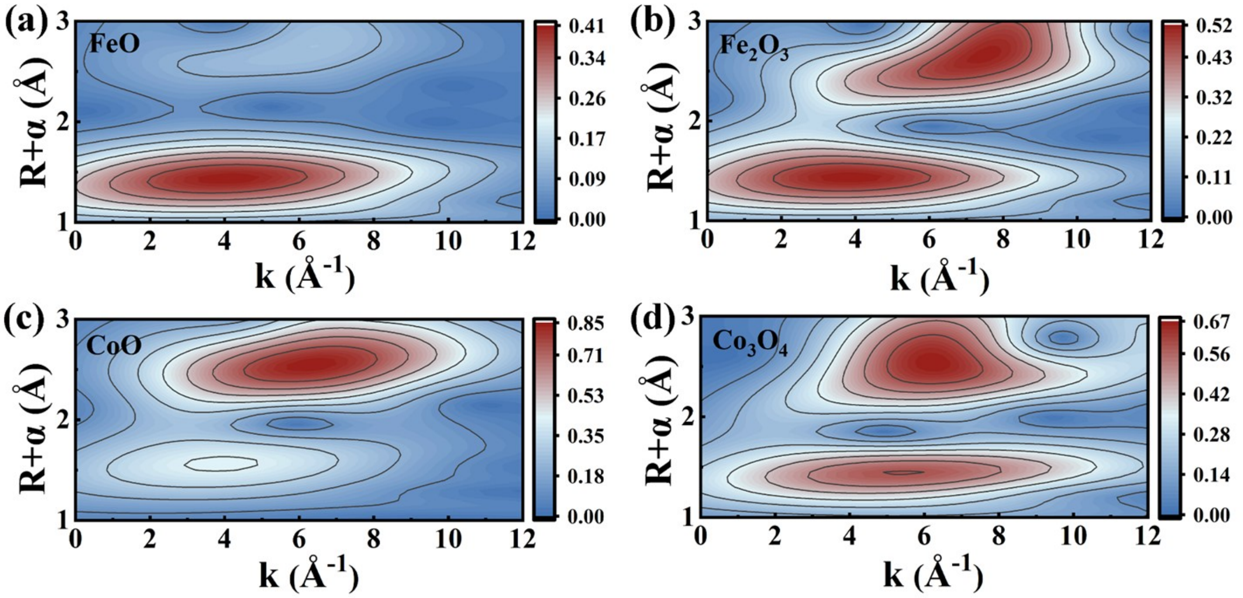

Figure S7. Wavelet transform (WT) mapping of (a) FeO, (b) Fe_2O_3, (c) CoO, (d) Co_3O_4.

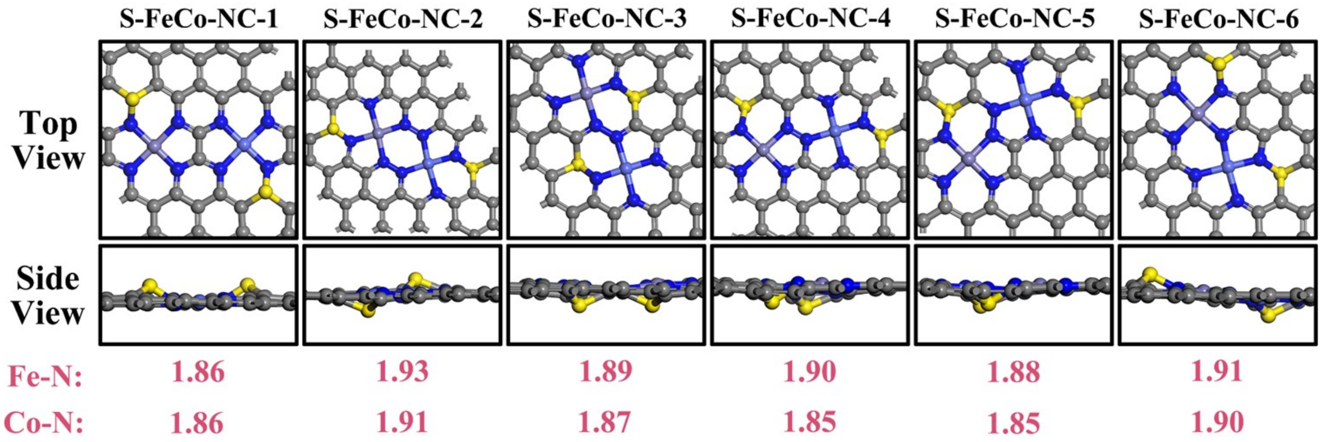

Figure S8. Atomic modeling of different types of metal sites in S-FeCo-NC and bond lengths of Fe-N/Co-N.

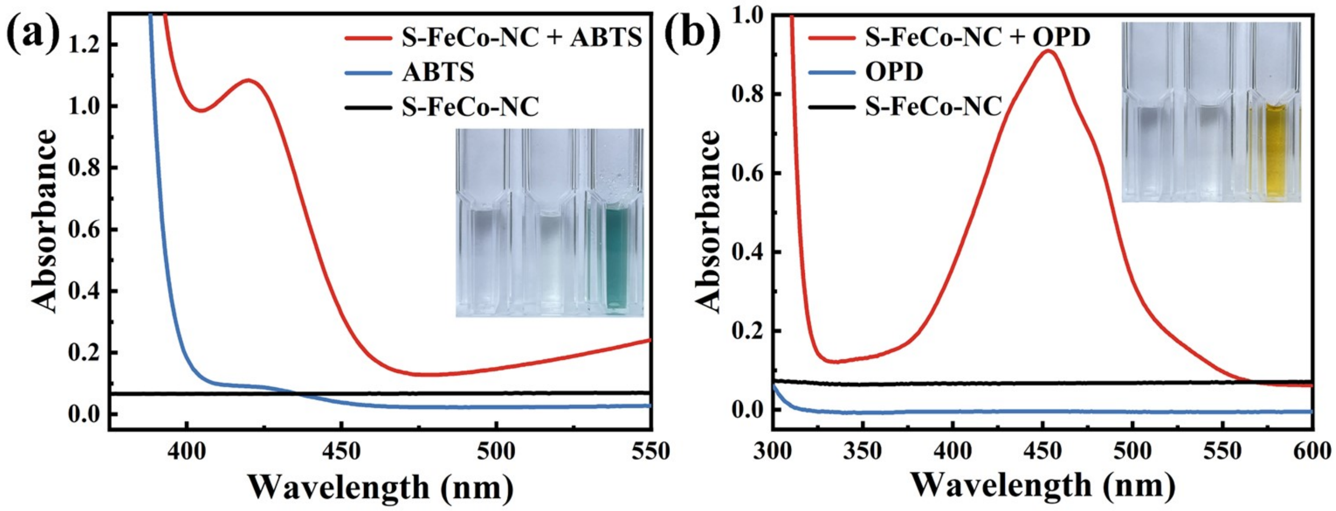

Figure S9. Enzyme catalytic feasibility experiments using (a) ABTS and (b) OPD as substrates, respectively.

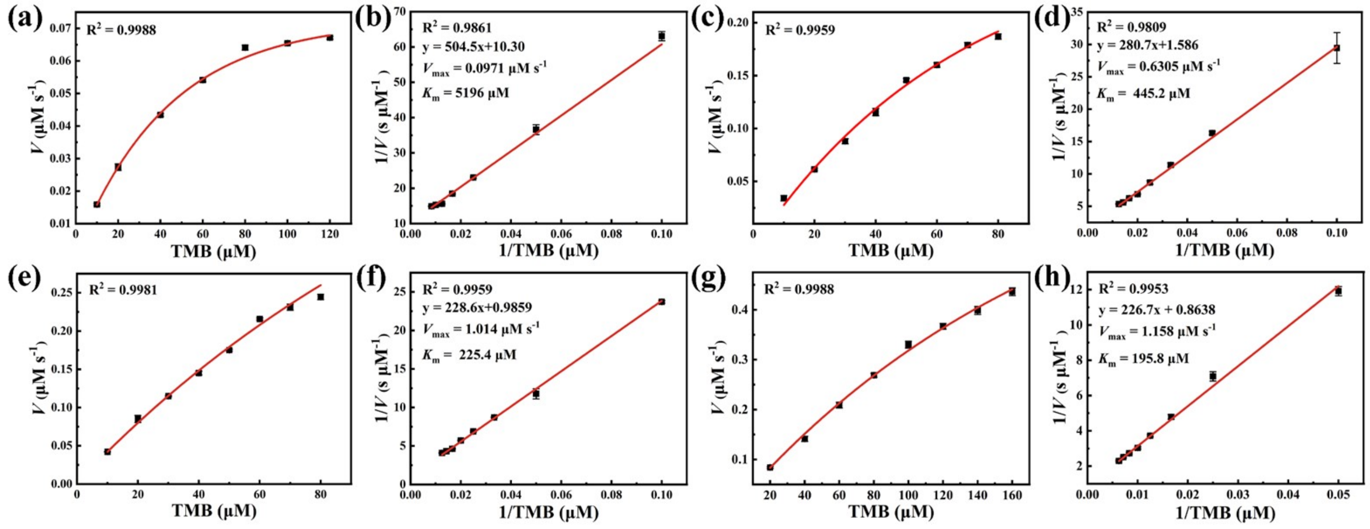

Figure S10. Michaelis-Menten curves for (a) Co-NC, (c) Fe-NC, (e) FeCo-NC, and (g) S-FeCo-NC. (b, d, f, and h) The corresponding Lineweaver-Burk plots.

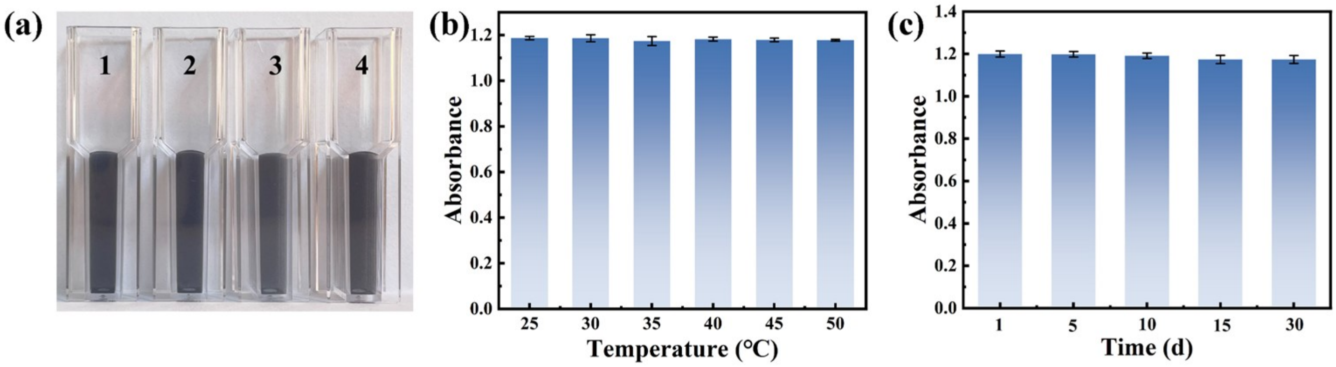

Figure S11. (1) Co-NC, (2) Fe-NC, (3) FeCo-NC, and (4) S-FeCo-NC uniformly dispersed aqueous solution (a). (b) Absorbance values of S-FeCo-NC oxidized TMB were detected at different temperatures. (c) Stability testing of S-FeCo-NC within thirty days of synthesis.

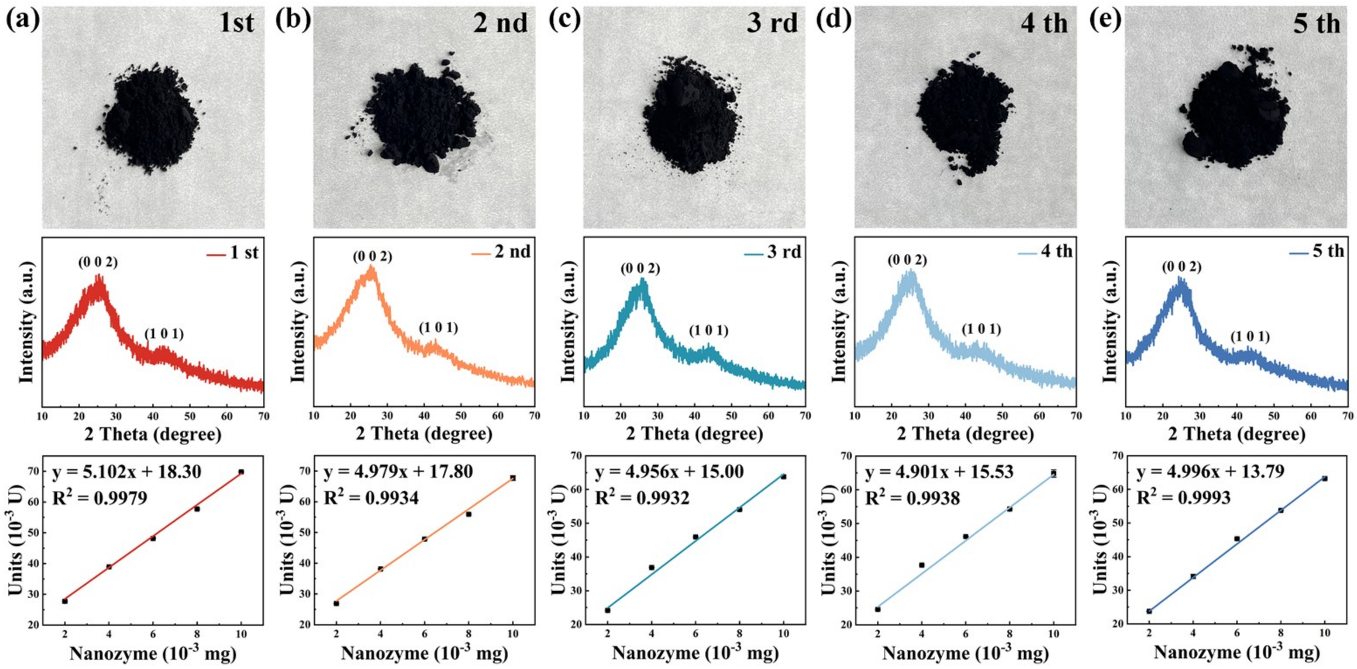

Figure S12. Powder morphology photographs, XPS spectra, and enzyme activity assessments across various batches of synthesized S-FeCo-NC materials. (a) 1st, (b) 2nd, (c) 3rd, (d) 4th, (e) 5th.

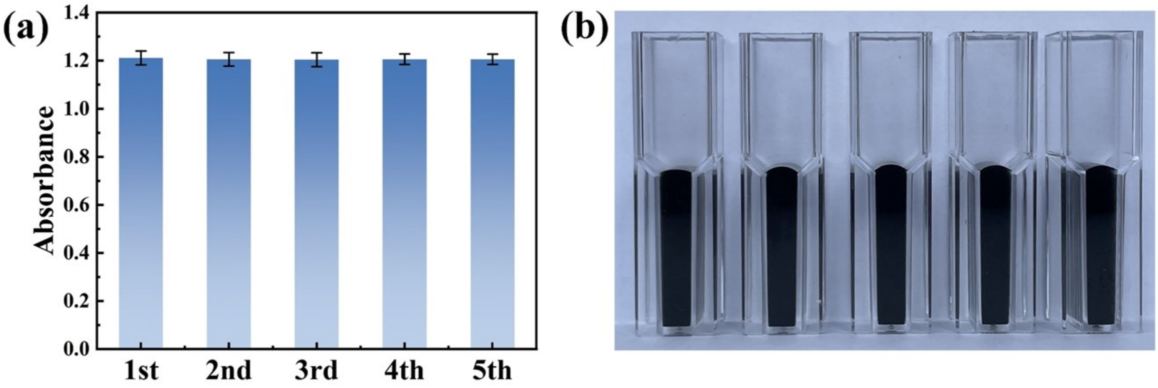

Figure S13. (a) Absorbance histograms under the same reaction conditions and (b) photographs of the material solutions of different batches of synthesized S-FeCo-NC materials.

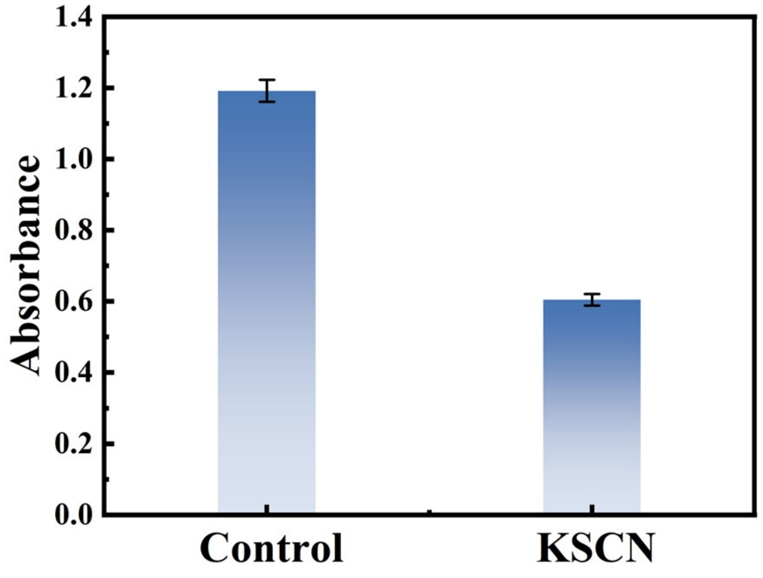

Figure S14. Metal active site toxicity test with KSCN concentration of 2 mM.

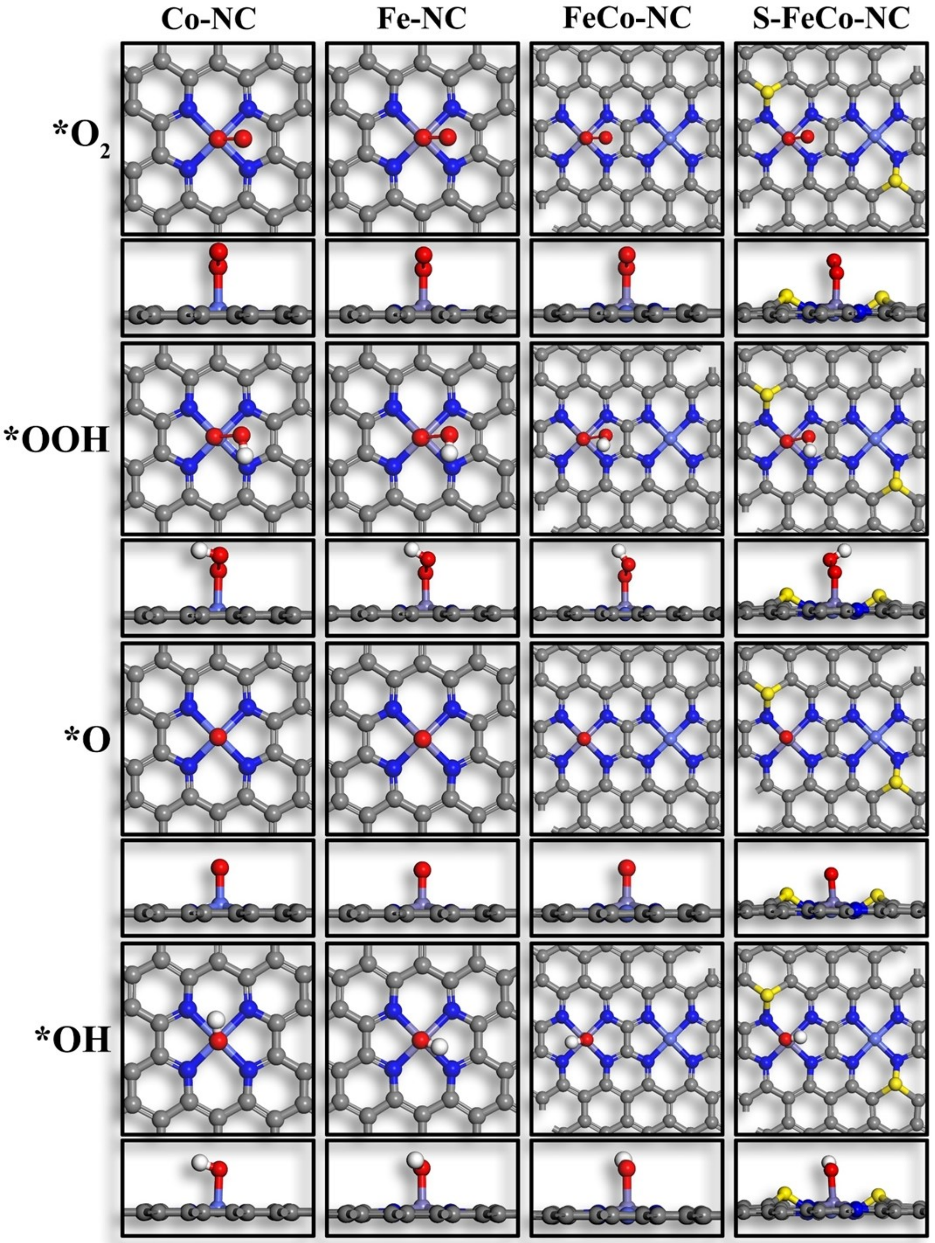

Figure S15. Adsorption optimization model for the 4-electron mechanism of the oxygen reduction reaction at each step.

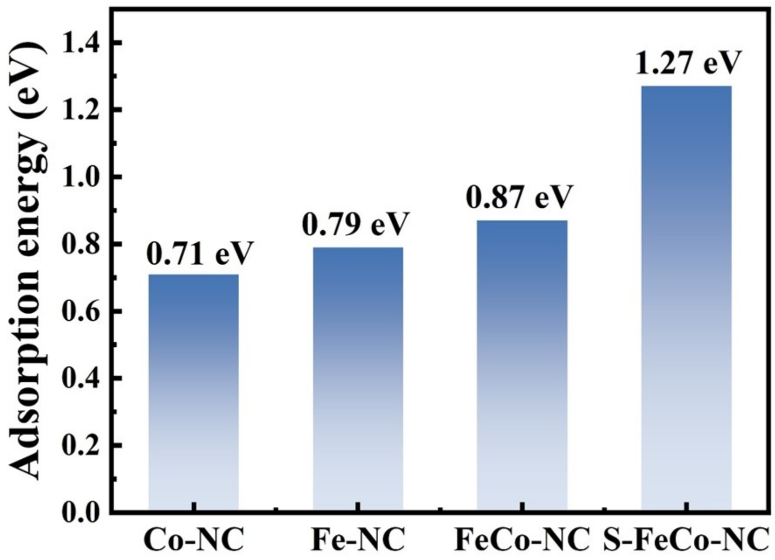

Figure S16. Oxygen adsorption energy of synthesized nanozymes.

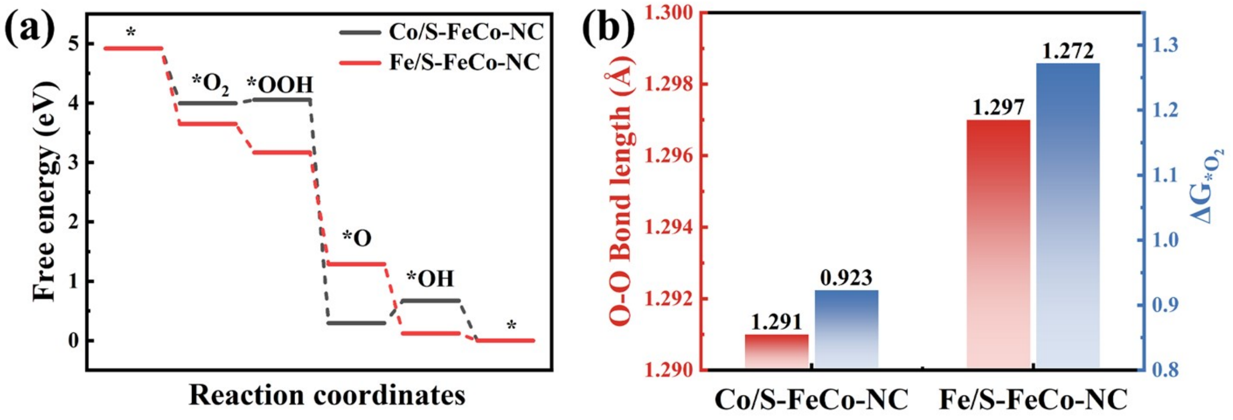

Figure S17. (a) Free energy step diagram of ORR catalytic mechanism using different metal active sites. (b) O-O bond lengths and O_2 adsorption energies (ΔG_{*O_2}) for O_2 adsorption using different metal active sites.

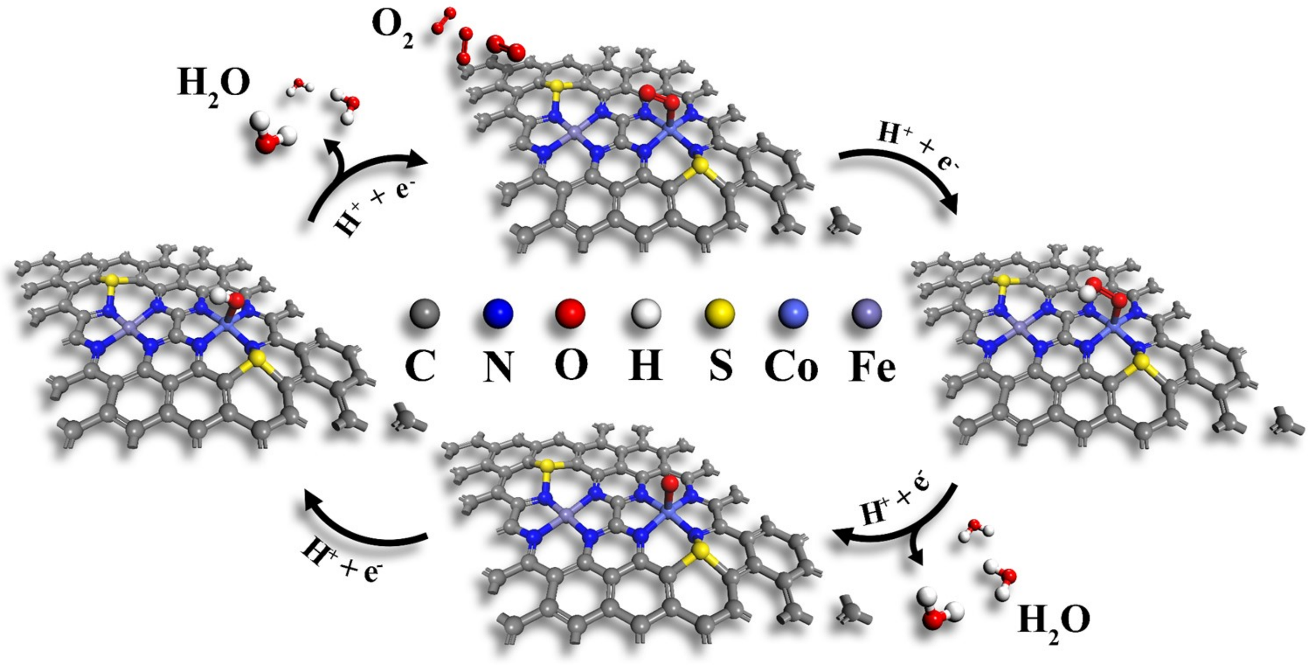

Figure S18. Mechanism of adsorption and desorption by 4e$^-$ pathways on Co atom adsorption sites.

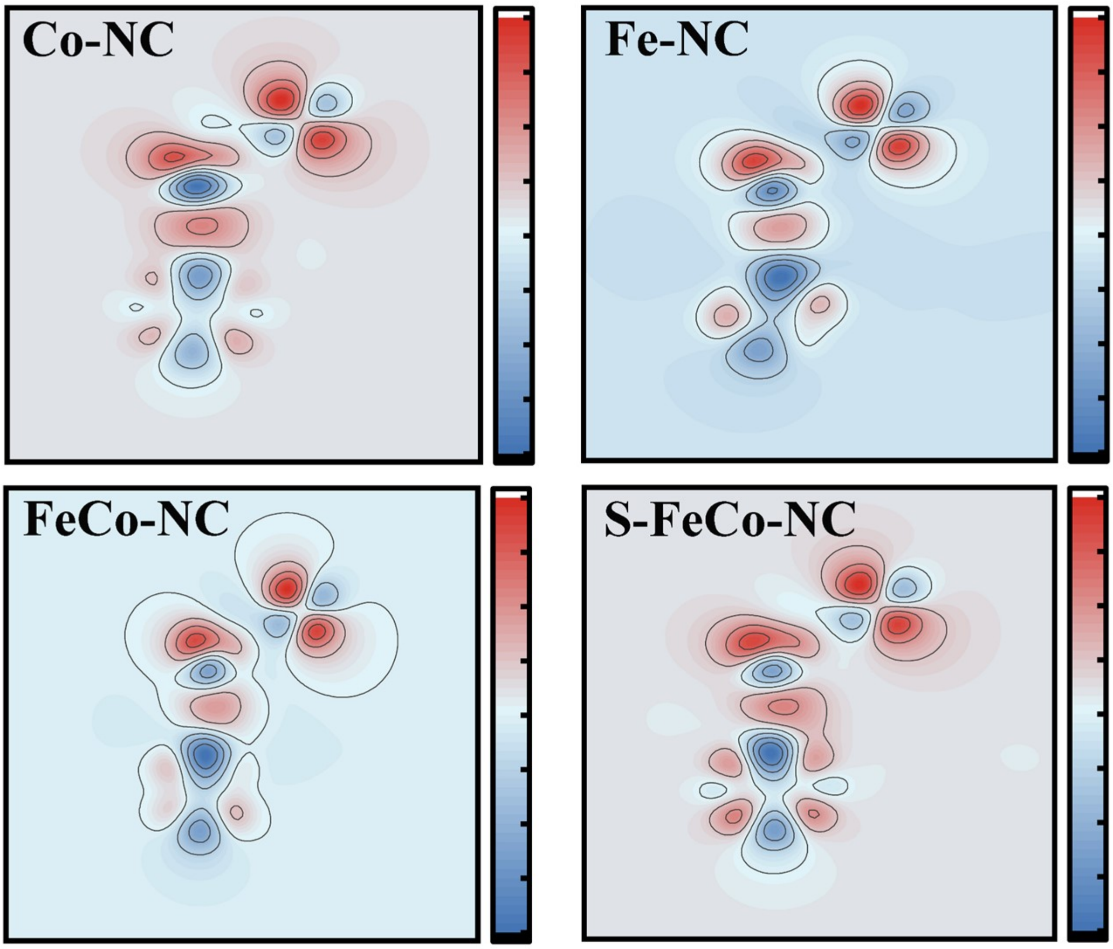

Figure S19. Differential charge density 2D images of adsorbed O_2 on four nanozymes, respectively.

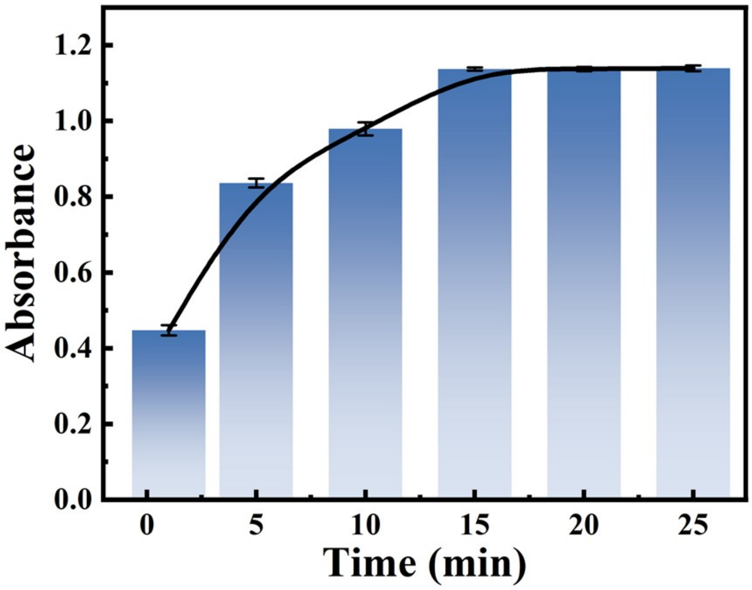

Figure S20. Absorbance changes of the S-FeCo-NC/AChE/TMB detection system at different times.

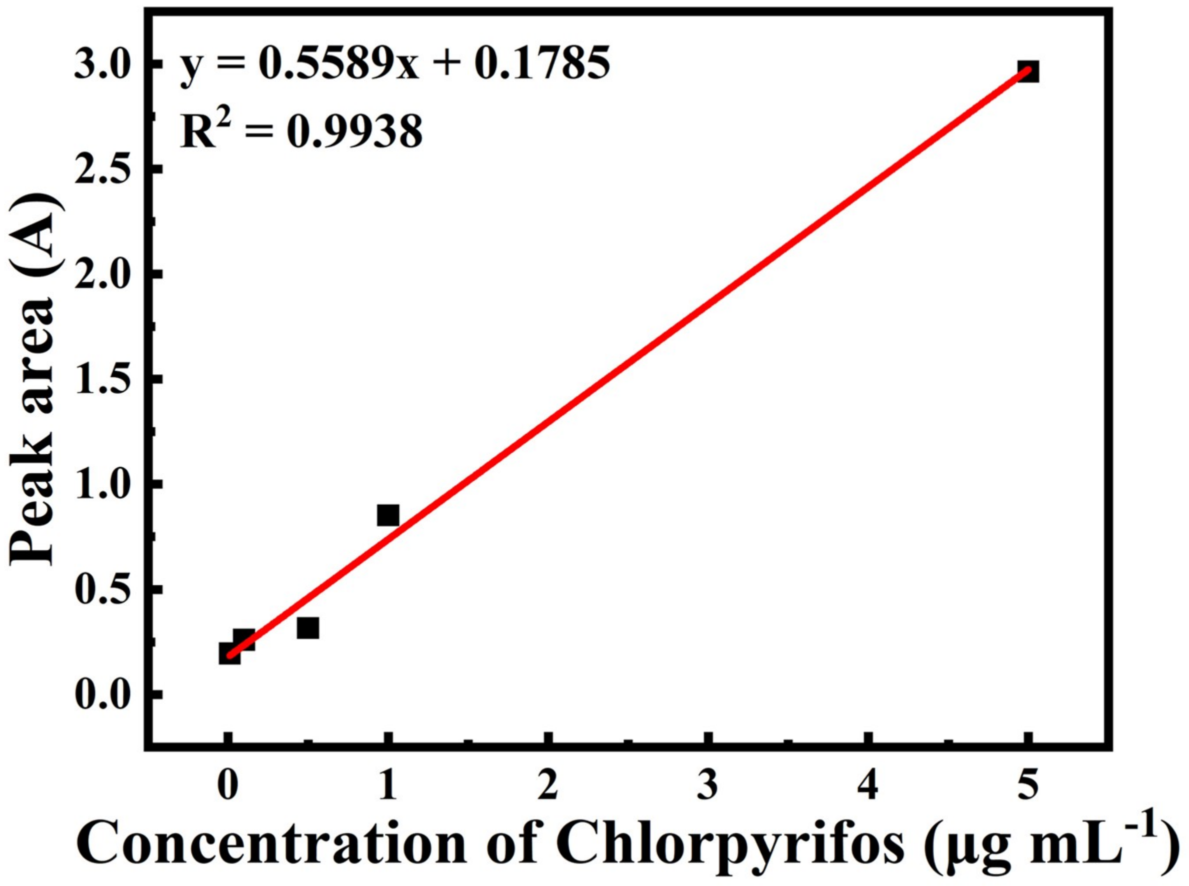

Figure S21. Standard curve for HPLC testing of chlorpyrifos concentrations.

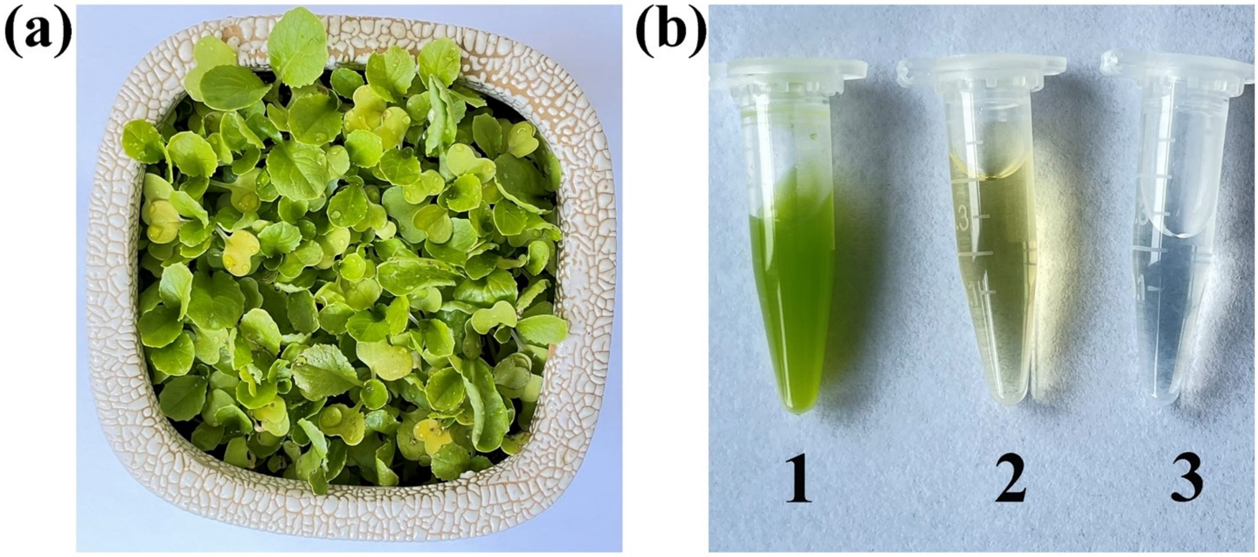

Figure S22. (a) Visible light image of Pak choi after 2 weeks of growth. (b) Visible images of the extracting solution of Pak choi (1, stock solution; 2, centrifugally filtered supernatant; 3, 50-fold diluted solution).

Table S1. EXAFS fitting parameters at the Fe K-edge for various S-FeCo-NC (S_0^2=0.75)

**Sample**	**shell**	**CN**	**R(Å)**	**σ^2**	**ΔE_0**	**R factor**
Fe foil	Fe-Fe	8	2.47±0.01	0.0048	6.4±0.8	0.0034
	Fe-Fe	6	2.85±0.01	0.0061		
S-FeCo-NC	Fe-N/O	3.5±0.4	1.86±0.02	0.0022	8.0±2.2	0.0097

Table S2. EXAFS fitting parameters at the Co K-edge for various S-FeCo-NC (S_0^2=0.77)

**Sample**	**shell**	**CN**	**R(Å)**	**σ^2**	**ΔE_0**	**R factor**
Co foil	Co-Co	12	2.49±0.01	0.0062	7.8±0.3	0.0013
S-FeCo-NC	Co-N/O	4.1±0.4	1.86±0.02	0.0057	3.6±2.3	0.0117

Table S3. Comparison of kinetic parameters between S-FeCo-NC and other single atom-based oxidase mimics.

**Catalyst**	K_m **(mM)**	V_{max} **(10^{-7} M s^{-1})**	W **(mg L^{-1})**	K_{cat} **(s^{-1})**	K_{cat}/K_m **(mM^{-1} s^{-1})**	**Refs.**
Fe-N-C SAzymes	1.81	0.006	17	0.35	1.93	1
Fe-N/C-CNTs	0.62	5.26	20	263	424.2	2
Ni-N/C-CNTs	0.28	0.245	20	12.25	43.75	
Co-N/C-CNTs	1.17	2.08	20	104	88.89	
Fe SAEs	0.13	0.225	5	45	346.2	3
Fe-N/C	0.94	5.98	20	299	318.1	4
FeBi-NC	0.11	3.19	10	319	2900	5
	0.21	0.70	2	350	1667	
Fe-N-C-400	0.27	3.38	500	6.76	25.04	6
Fe-N-C-800	0.23	1.33	500	2.66	11.57	
Zn-N-C	0.22	1.07	215	4.97	22.59	7
$Co(OH)_2$ nanocages	0.362	0.606	-	-	-	8
CoNPs/MC	0.39	1.340	15	89.3	228.9	9
CoO/CeO_2	1.81	0.27	80	3.37	1.86	10
$Co_2V_2O_7$	0.311	0.258	70	3.69	11.86	11
$Co@Fe_3O_4$	1.17	3.79	200	18.9	16.15	12
$CoPW_{11}O_{39}$	0.81	0.13	60	2.17	2.68	13
SA Co-MoS_2	3.349	6.49	30	216	64.50	14
$CdCo_2O_4$ nanosheets	0.317	0.219	200	1.09	3.44	15
**Co-NC**	**5.20**	**0.97**	**20**	**48.5**	**9.33**	**This work**
**Fe-NC**	**0.45**	**6.31**	**20**	**316**	**702.2**	
**FeCo-NC**	**0.23**	**10.14**	**20**	**507**	**2204**	
**S-FeCo-NC**	**0.20**	**11.58**	**20**	**579**	**2895**	

Table S4. Comparative table for the detection of AChE.

**Method**	**Samples**	**Linear range ($mU\ mL^{-1}$)**	**LOD ($mU\ mL^{-1}$)**	**Reference**
Luminescence	Pd @AuNR	0-80	18.5	16
Fluorescence	PhO-dex-GO	0.1-100	0.27	17
Fluorescence	Carbon dots	14.2-121.8	4.25	18
Fluorescence	PAA-CeO2	0.263-50	0.263	19
Fluorescence	Perylene probe/MnO_2 NS	5-100	2.5	20
Colorimetry	Citrate-CeO2	0-1400	3.5	21
Colorimetry	Fe-SAs/NC	2-70	0.56	22
**Colorimetry**	**S-FeCo-NC**	**0.05-0.5**	**0.02**	**This work**

Table S5. OPs assay performance of our strategy and other methods.

Method	OPs	Samples	Liner range ($ng\ mL^{-1}$)	LOD ($ng\ mL^{-1}$)	Reference
Colorimetric	paraoxon	MnO2	1-100	1	23
Colorimetric	malathion	Pd@ AuNR	60-200000	60	16
Colorimetric	omethoate	AuNPs	21.32-2132	21.32	24
Colorimetric	parathion	Au3+-CTAB	35-1290	35	25
Fluorescence	dimethoate	TPE-1	9-22500	8	26
Fluorescence	chlorpyrifos	C-dots	10-1000	3	27
Colorimetric	chlorpyrifos	Fe-N-C SAzymes	0.1-10	0.97	1
Fluorescence	paraoxon	ZIF-8/MB	20-4000	1.7	28
Colorimetric	paraoxon-ethyl	Cu SASCs	1-300	0.6	29
Colorimetric	paraoxon-ethyl	Fe SASCs	0.1-10	0.97	30
Colorimetric	paraoxon	FeSNC	1-100	0.87	31
**Colorimetric**	**chlorpyrifos**	**S-FeCo-NC**	**1-4**	**0.2**	**This work**

Table S6. Repeat detection experiments of acetylcholinesterase using the sensing platform over five days.

Time (d)	Added (mU mL^{-1})	ΔA (652 nm)	Average	RSD (%)	Deviation
1	0.200	0.134			
2	0.200	0.138			
3	0.200	0.137	0.138 ± 0.0029	2.10 %	0.006
4	0.200	0.142			
5	0.200	0.139			

Table S7. Repeat detection experiments of chlorpyrifos using the sensing platform over five days.

Time (d)	Added (ng mL^{-1})	Abs. (652 nm)	Average	RSD (%)	Deviation
1	2.00	0.244			
2	2.00	0.229			
3	2.00	0.223	0.232 ± 0.011	4.74 %	0.001
4	2.00	0.221			
5	2.00	0.245			

Table S8. Comparison between this system and HPLC standard method testing

Method	Measured (ng mL^{-1})	Average	RSD (%)	Deviation	RSD (%)
HPLC	3.50	3.68	4.56	0.08	2.15
	3.83				
	3.72				
This work	3.91	3.57	8.91		
	3.28				
	3.52				

Table S9. Recoveries and RSDs of paraoxon based on our sensor.

	Added (ng mL^{-1})	Measured (ng mL^{-1})	Recovery (%)	RSD (%)
1	1.50	1.53 ± 0.041	102.00	2.67
2	3.00	2.99 ± 0.030	99.67	3.02
3	4.00	4.06 ± 0.368	101.50	9.07

Reference

1 Y. Wu, L. Jiao, X. Luo, W. Xu, X. Wei, H. Wang, H. Yan, W. Gu, B. Z. Xu, D. Du, Y. Lin and C. Zhu, *Small*, 2019, **15**, 1903108.

2 Y. Wang, Z. Zhang, G. Jia, L. Zheng, J. Zhao and X. Cui, *Chem. Commun.*, 2019, **55**, 5271-5274.

3 C. Zhao, C. Xiong, X. Liu, M. Qiao, Z. Li, T. Yuan, J. Wang, Y. Qu, X. Wang, F. Zhou, Q. Xu, S. Wang, M. Chen, W. Wang, Y. Li, T. Yao, Y. Wu and Y. Li, *Chem. Commun.*, 2019, **55**, 2285-2288.

4 Q. Chen, S. Li, Y. Liu, X. Zhang, Y. Tang, H. Chai and Y. Huang, *Sens. Actuators B: Chem.*, 2020, **305**, 127511.

5 Q. Chen, Y. Liu, Y. Lu, Y. Hou, X. Zhang, W. Shi and Y. Huang, *J. Hazard. Mater.*, 2022, **422**, 126929.

6 Y. Xu, J. Xue, Q. Zhou, Y. Zheng, X. Chen, S. Liu, Y. Shen and Y. Zhang, *Angew. Chem. Int. Ed.*, 2020, **59**, 14498-14503.

7 B. Xu, H. Wang, W. Wang, L. Gao, S. Li, X. Pan, H. Wang, H. Yang, X. Meng, Q. Wu, L. Zheng, S. Chen, X. Shi, K. Fan, X. Yan and H. Liu, *Angew. Chem. Int. Ed.*, 2019, **58**, 4911-4916.

8 H. Zhu, Z. Quan, H. Hou, Y. Cai, W. Liu and Y. Liu, *Anal. Chim. Acta*, 2020, **1132**, 101-109.

9 W. Dong, Y. Zhuang, S. Li, X. Zhang, H. Chai and Y. Huang, *Sens. Actuators B: Chem.*, 2018, **255**, 2050-2057.

10 J. Ge, X. Yang, J. Luo, J. Ma, Y. Zou, J. Li, W. Luo, X. Cheng and Y. Deng, *Appl. Mater. Today*, 2019, **15**, 482-493.

11 X. Zhang, G. Han, R. Zhang, Z. Huang, H. Shen, P. Su, J. Song and Y. Yang, *ACS Appl. Bio Mater.*, 2020, **3**, 1469-1480.

12 Y. Wang, H. Li, L. Guo, Q. Jiang and F. Liu, *RSC Adv.*, 2019, **9**, 18815-18822.

13 Y. He, X. Li, X. Xu, J. Pan and X. Niu, *J. Mater. Chem. B*, 2018, **6**, 5750-5755.

14 Y. Wang, K. Qi, S. Yu, G. Jia, Z. Cheng, L. Zheng, Q. Wu, Q. Bao, Q. Wang, J. Zhao, X. Cui and W. Zheng, *Nano-Micro Letters*, 2019, **11**, 102.

15 X. Wei, J. Chen, M. C. Ali, J. C. Munyemana and H. Qiu, *Microchim. Acta*, 2020, **187**, 314.

16 S. Singh, P. Tripathi, N. Kumar and S. Nara, *Biosens. Bioelectron.*, 2017, **92**, 280-286.

17 T. W. Kang, S.-J. Jeon, H.-I. Kim, J. H. Park, D. Yim, H.-R. Lee, J.-M. Ju, M.-J. Kim and J.-H. Kim, *ACS Nano*, 2016, **10**, 5346-5353.

18 Z. Qian, L. Chai, C. Tang, Y. Huang, J. Chen and H. Feng, *Sens. Actuators B: Chem.*, 2016, **222**, 879-886.

19 S.-X. Zhang, S.-F. Xue, J. Deng, M. Zhang, G. Shi and T. Zhou, *Biosens. Bioelectron.*, 2016, **85**, 457-463.

20 Y. Zhang, C. Zhang, J. Chen, Y. Li, M. Yang, H. Zhou, S. A. Shahzad, H. Qi, C. Yu and S. Jiang, *J. Mater. Chem. C*, 2017, **5**, 4691-4694.

21 H. Cheng, S. Lin, F. Muhammad, Y.-W. Lin and H. Wei, *ACS Sens.*, 2016, **1**, 1336-1343.

22 M. Wang, L. Liu, X. Xie, X. Zhou, Z. Lin and X. Su, *Sens. Actuators B: Chem.*, 2020, **313**, 128023.

23 X. Yan, Y. Song, X. Wu, C. Zhu, X. Su, D. Du and Y. Lin, *Nanoscale*, 2017, **9**, 2317-2323.

24 P. Wang, Y. Wan, A. Ali, S. Deng, Y. Su, C. Fan and S. Yang, *Sci. China Chem.*, 2015, **59**, 237-242.

25 S. Wu, D. Li, J. Wang, Y. Zhao, S. Dong and X. Wang, *Sens. Actuators B: Chem.*, 2017, **238**, 427-433.

26 Y. Cai, J. Fang, B. Wang, F. Zhang, G. Shao and Y. Liu, *Sens. Actuators B: Chem.*, 2019, **292**, 156-163.

27 B. Lin, Y. Yan, M. Guo, Y. Cao, Y. Yu, T. Zhang, Y. Huang and D. Wu, *Food Chem.*, 2018, **245**, 1176-1182.

28 X. Li, X. Gao, P. Gai, X. Liu and F. Li, *Sens. Actuators B: Chem.*, 2020, **323**, 128701.

29 Y. Wu, J. Wu, L. Jiao, W. Xu, H. Wang, X. Wei, W. Gu, G. Ren, N. Zhang, Q. Zhang, L. Huang, L. Gu and C. Zhu, *Anal. Chem.*, 2020, **92**, 3373-3379.

30 Y. Wu, L. Jiao, W. Xu, W. Gu, C. Zhu, D. Du and Y. Lin, *Small*, 2019, **15**, 1900632.

31 L. Jiao, Y. Kang, Y. Chen, N. Wu, Y. Wu, W. Xu, X. Wei, H. Wang, W. Gu, L. Zheng, W. Song and C. Zhu, *Nano Today*, 2021, **40**, 101261.
